# MiR-146a alleviates inflammatory bowel disease in mice through systematic regulation of multiple genetic networks

**DOI:** 10.3389/fimmu.2024.1366319

**Published:** 2024-05-10

**Authors:** Fengting Zhu, Taotan Yang, Mengmeng Ning, Yang Liu, Wei Xia, Yan Fu, Ting Wen, Mei Zheng, Ruilong Xia, Ran Qian, Yang Li, Minxuan Sun, Jianping Liu, Li Tian, Qian Zhou, Xin Yu, Changgeng Peng

**Affiliations:** ^1^ The First Rehabilitation Hospital of Shanghai, Clinic Center for Brain and Spinal Cord Research, School of Medicine and Advanced Institute of Translational Medicine, Tongji University, Shanghai, China; ^2^ Pre-clinical College, Dali University, Dali, Yunnan, China; ^3^ Xiang-Xing College, Hunan University of Traditional Chinese Medicine, Changsha, China; ^4^ The Third Xiangya Hospital, Central South University, Changsha, Hunan, China; ^5^ Department of Clinical Laboratory, Shanghai Songjiang District Central Hospital, Shanghai, China; ^6^ Suzhou Institute of Biomedical Engineering and Technology, Chinese Academy of Sciences, Suzhou, China; ^7^ School of Biomedical Engineering (Suzhou), Division of Life Sciences and Medicine, University of Science and Technology of China, Hefei, China; ^8^ Jiangxi Provincial Key Laboratory of Digestive Diseases, Department of Gastroenterology, The First Affiliated Hospital, Jiangxi Medical College, Nanchang University, Nanchang, Jiangxi, China

**Keywords:** *miR-146a-5p*, *miR-146a-3p*, inflammatory bowel disease, genetic regulatory networks, MMPs, chemokines, cytokines, therapy

## Abstract

**Introduction:**

Inflammatory bowel disease (IBD) is a chronic disease involving multiple genes, and the current available targeted drugs for IBD only deliver moderate efficacy. Whether there is a single gene that systematically regulates IBD is not yet known. *MiR-146a* plays a pivotal role in repression of innate immunity, but its function in the intestinal inflammation is sort of controversy, and the genetic regulatory networks regulated by miR-146a in IBD has not been revealed.

**Methods:**

RT-qPCR was employed to detect the expression of *miR-146a* in IBD patients and in a mouse IBD model induced by dextran sulfate sodium (DSS), and then we generated a *miR-146a* knock-out mouse line with C57/Bl6N background. The disease activity index was scored in DSS-treated miR-146a deficiency mice and their wild type (*WT*) littermates. Bulk RNA-sequencing, RT-qPCR and immunostaining were done to illustrate the downstream genetic regulatory networks of *miR-146a* in flamed colon. Finally, the modified *miR-146a* mimics were used to treat DSS-induced IBD in *miR-146a* knock-out and *WT* IBD mice.

**Results:**

We showed that the expression of *miR-146a* in the colon was elevated in dextran sulfate sodium (DSS)-induced IBD mice and patients with IBD. DSS induced dramatic body weight loss and more significant rectal bleeding, shorter colon length, and colitis in *miR-146a* knock-out mice than *WT* mice. The miR-146a mimics alleviated DSS-induced symptoms in both *miR-146a^-/-^* and *WT* mice. Further RNA sequencing illustrated that the deficiency of *miR-146a* de-repressed majority of DSS-induced IBD-related genes that cover multiple genetic regulatory networks in IBD, and supplementation with *miR-146a* mimics inhibited the expression of many IBD-related genes. Quantitative RT-PCR or immunostaining confirmed that *Ccl3, Saa3, Csf3, Lcn2, Serpine1, Serpine2*, MMP3, MMP8, MMP10, IL1A, IL1B, IL6, CXCL2, CXCL3, S100A8, S100A9, TRAF6, P65, p-P65, and IRAK1 were regulated by miR-146a in DSS induced IBD. Among them, *MMP3, MMP10, IL6, IL1B, S100A8, S100A9, SERPINE1, CSF3*, and *IL1A* were involved in the active stage of IBD in humans.

**Discussion:**

Our date demonstrated that miR-146a acts as a top regulator in C57/BL6N mice to systematically repress multiple genetic regulatory networks involved in immune response of intestine to environment factors, and combinatory treatment with *miR-146a-5p* and *miR-146a-3p* mimics attenuates DSS-induced IBD in mice through down-regulating multiple genetic regulatory networks which were increased in colon tissue from IBD patients. Our findings suggests that *miR-146a* is a top inhibitor of IBD, and that *miR-146a-5p* and *miR-146a-3p* mimics might be potential drug for IBD.

## Introduction

Genetic susceptibility and environment are the two main risk factors for inflammatory bowel disease (IBD) that affects approximately 1% of the population (1, 2). IBD mainly includes Crohn’s disease (CD) and ulcerative colitis (UC) (1, 3, 4). During the development of IBD, continuous inflammation progressively causes damage of intestinal epithelial barrier and vascular endothelium, and consequently affects digestion and absorption and leads to rectal bleeding and body weight loss (1, 5). The progression of IBD is known to be promoted by upregulated genetic regulatory networks of chemokines, cytokines, other pro-inflammatory factors (S100A8/A9, CSF3, etc.), extracellular matrix breakdown enzymes, and coagulation (2, 5–13). Because of the involvement of multiple genetic regulatory networks, IBD is a complicated and heterogeneous disease and remains difficult to cure. Although conventional drugs such as 5-aminosalicylates, corticosteroids, and immunomodulators have limited efficacy and unavoidable side effects, they are still used as first-line agents for the treatment of mild to moderate IBD (3, 14). Even recently developed drugs, such as antibodies against TNFα, α4β7 integrins, and interleukins, as well as inhibitors of Janus kinase, confer only 30% to 60% efficacy for remission of IBD in clinical trials (3, 15, 16). The reason for the limited efficacy of IBD drugs is the fact that these drugs can only inhibit one genetic regulatory network of IBD; thus, discovering top regulators of IBD is necessary for developing new drugs to target all genetic regulatory networks involved in IBD to obtain higher efficacy.

MicroRNA (miRNA) can regulate hundreds to even up to thousands of direct target genes; therefore, it could be a top regulator for chronic diseases with multiple genes involved. For example, the *miR-183-96-182* cluster regulates approximately 80% of genes in genetic regulatory network of peripheral injury-induced neuropathic pain, including the sodium channel network and the calcium channel network (17–19). It is known that *miR-146a* regulates innate immune responses through repressing two targets, IL-1 receptor-associated kinase 1 (IRAK1) and TNF receptor-associated factor 6 (TRAF6), in a negative feedback manner (20–24), and that *miR-146a*-deficient mice with a 129.B6, but not C57BL/6, genetic background have an elevated serum level of IL6 and develop aging-related autoimmune diseases with multiorgan (liver, kidneys, and lungs) inflammation (25). However, the role of *miRNA-146a* in intestinal inflammation is controversial (26, 27), and all genetic regulatory networks regulated by *miR-146a* and the function of *miR-146a* in IBD have not been fully uncovered. Here, we showed that *miR-146a-*deficient mice developed severe IBD after induction of dextran sulfate sodium (DSS) through de-repression of multiple genetic regulatory networks, and the combination of the modified *miR-146a-5p* and *miR-146a-3p* mimics (and not when administered on their own) can attenuate IBD symptoms in both *miR-146a*-null and *WT* mice via repressing the genetic regulatory networks.

## Materials and methods

### Animals

In this study, all animals were housed at 21°C with 50% humidity, on a 12-h light:12-h dark schedule in the standard animal facility, five per cage, in accordance with the guidelines of Tongji University, and all animal work was conducted under ethical permission from the Tongji University ethical review panel.

*MiR-146a^+/-^* mice were generated by Cyagen Biosciences Inc. (Guangzhou, China) using CRISPR/Cas9 technology. Briefly, *in vitro* transcribed Cas9 mRNA and gRNA (gRNA1: TGCACCACCCATGATAGGGCAGG, gRNA2: GCTTCGCTTTTCCCAACCGGAGG) were injected into fertilized eggs of C57BL/6N mice. PCR product sequencing using two pairs of primers (*miR-146a* F1, 5′ AAGGGAAGGATTGAACATGACACA 3′, *miR-146a* R1 5′ TTATTGCCTCTCTACAAGGACCTG 3′, and *miR-146a* R2 5′ ACCATCAATAGCAGAGATGACTGG 3′) was performed to identify F0-generation mice carrying a *miR-146a* knock-out allele. The positive F0-generation mice were then mated with wild-type C57BL/6N mice to obtain F1-generation mice, and the F1-generation *miR-146a^+/-^* mice were identified by PCR, and Sanger sequencing confirmed a deletion of 1,467 bp, which did not affect the gene sequence of the gene Gm12148 which is close to *miR-146a*. Male and female experimental *WT* C57BL/6N mice were purchased from Vital River Laboratory Animal Technology Co., Ltd. (Zhejiang, China).

### Collection of human samples

During colonoscopy, biopsy samples were collected from 22 adult patients with ongoing gut symptoms by the Department of Gastroenterology, The Third Xiangya Hospital, Central South University according to the ethics permit (R19057) approved by the Ethics Committee of The Third Xiangya Hospital. All fresh samples from patients who requested biopsy examination and agreed to donate biopsy tissue for our research were included in this study, with no exclusion criteria. Among them, six subjects were without IBD (non-IBD), and seven and nine patients were with IBD in the active stage (active IBD) or inactive stage (inactive IBD). The information of patients with IBD is provided in **Table S1**. Biopsy samples were immediately frozen in dry ice and stored in a −80°C freezer until further analysis.

### DSS-induced experimental colitis and treatment

Experiment 1: 2% DSS (molecular weight of 36–50 kDa, MP Biomedicals, Irvine, CA, USA) in water was used to induce C57BL/6N mice and *miR-146a^+/-^* mice to become IBD models. *WT* and *miR-146a^+/-^* mice were randomly divided into four groups: *WT* control group, *miR-146a^+/-^* control group, DSS*-*treated *WT* group, and DSS*-*treated *miR-146a^+/-^* group. Control group mice had free access to tap water, and DSS group mice and three additional *miR-146a^-/-^* mice were given 2% DSS in drinking water for 6 days to induce experimental colitis and fed with water on day 7. Mice were sacrificed on day 7 and samples were collected.

Experiment 2: To investigate the efficacy of *miR-146a* mimics in DSS-induced *miR-146a^-/-^* IBD mice, *miR-146a^-/-^* mice were randomly divided into three groups: saline control group, saline + DSS group, and *miR-146a* mimics + DSS group. Saline control group mice had free access to tap water and treated with vehicle saline. Saline + DSS group and *miR-146a* mimics + DSS group mice were given 2.5% DSS (a new batch from the same supplier) in drinking water for 7 days to induce experimental colitis; meanwhile, saline and *miR-146a* mimics [the modified *miR-146a*-*5p* mimics (40 pg per gram of body weight) and *miR-146a*-*3p* mimics (80 pg per gram of body weight)] were purchased from Suzhou Biosyntech Co. Suzhou, China (**Supplementary Table S2**) and administrated via gavage and intraperitoneal injection every other day from day 1 for five times, respectively. Mice were sacrificed on day 9 and samples were collected.

Experiment 3: To examine the efficacy of *miR-146a* mimics in DSS-induced *WT* mice, C57BL/6N mice were randomly divided into three groups: saline control group, saline + DSS group, and *miR-146a* mimics + DSS group. Saline control group mice had free access to tap water and treated with vehicle saline. Saline + DSS group and *miR-146a* mimics + DSS group mice were fed with 2.5% DSS containing water on day 1 and followed with 2% DSS containing water from day 2 to day 6, and treated with vehicle saline and *miR-146a* mimics [the modified *miR-146a*-*5p* mimics (6.5 pg per g body weight) and *miR-146a*-*3p* mimics (3.25 pg per g body weight)], respectively, via intraperitoneal injection every other day from day 1 for five times. Mice were sacrificed on day 9 and samples were collected.

### Evaluation of the disease activity index

The disease activity index (DAI) was determined by weight loss, stool consistency, and fecal occult blood.

The weight loss score is as follows: 0 points, <1%; 1 point, 1%–5%; 2 points, 5%–10%; 3 points, 10%–20%; and 4 points, >20%. The stool consistency is divided into three conditions: 0 points, normal stool; 2 points, loose stool; and 4 points, water-like stool. The feces was stained with the fecal occult blood test kit (Zhuhai BaSO Biotechnology Co., Zhuhai, China), and the fecal occult blood was scored according to the following scale: 0 points, negative; 2 points, positive; and 4 points, obvious blood on feces. Note that spouted rectal bleeding, which was indicated by bloodstain on mouse tail, was not counted in the score of fecal occult blood.

### Collection of mouse colon tissues and measurement

Colons were quickly removed from mice, and their length was measured and photographed. After removing the content, the colon was washed twice with cold saline. Dissected colon tissues were snap-frozen and stored at −80°C for RT-qPCR detection. For immunofluorescence and histochemical staining detection, colons were immersed in 4% PFA overnight and embedded in paraffin after progressive dehydration.

### RNA sequencing

Total RNA was isolated from colons using Trizol Reagent (Thermo Fisher Scientific, Massachusetts, USA) and 1 µg of total RNA was used for library preparation according to Illumina standard instruction (VAHTS Universal V6 RNA-seq Library Prep Kit for Illumina^®^). An Agilent 4200 bioanalyzer was employed to evaluate the concentration and size distribution of cDNA library before sequencing with an Illumina novaseq6000. The protocol of high-throughput sequencing was followed according to the manufacturer’s instructions (Illumina). The raw reads were filtered by Seqtk before mapping to genome using Hisat2 (version: 2.0.4) (28). The gene fragments were counted using stringtie (v1.3.3b) followed by TMM (trimmed mean of M values) normalization (29–31). Significant differentially expressed genes (DEGs) were identified as those with a *p* value < 0.01 and fold change >2 using the edgeR software (32). The RNA-seq raw data as well as gene read counts for individual samples are accessible at Gene Expression Omnibus (GEO) under accession number GSE247433.

### Real-time quantitative RT-PCR

Total RNA was extracted from mouse colons and human samples using Trizol reagent (Thermo Fisher Science, Massachusetts, USA) and 2 μg of total RNA was reverse-transcribed with the Revert Aid First Strand Gene Synthesis Kit (Thermo Fisher Science, Massachusetts, USA). Using SYB-Green and the ABI QuantStudio 3 machine (Thermo Fisher Scientific, Massachusetts, USA), the expression level of *Mmp3*, *Mmp8*, *Mmp10*, *Il6*, *Il1a*, *Il1b*, *Cxcl2*, *Cxcl3*, *S100a8*, *S100a9*, *Lcn2*, *Serpine1*, *Serpine2*, *Ccl3*, *Saa3*, and *Csf3* was quantified by real-time PCR with specific primers listed in **Supplementary Table S3** and normalized to *Gapdh* RNA. For miRNA detection, 1 μg of total RNA was polyadenylated and reverse-transcribed using the Catch-All™ miRNA & mRNA RT-PCR Kit (Pengekiphen, Suzhou, China) according to the manufacturer’s instructions. Subsequently, qRT-PCR assays were conducted using the Catch-All™ miRNA & mRNA universal PCR primer as the reverse primer, and the specific miRNA forward primers listed in **Supplementary Table S3** or as previously described (18). The amplification conditions were as follows: an initial step at 95°C for 10 min, and 40 cycles of 15 s at 95°C and 1 min at 60°C. All assays were performed in triplicate and negative controls were included by omitting the template. The Ct value was recorded for each reaction, and the expression level of miRNAs was calculated relative to U6, a ubiquitously expressed snRNA.

### Hematoxylin and eosin staining

Paraffin sections with a thickness of 4 µm were stained using a hematoxylin and eosin (HE) staining kit (Solarbio Life Sciences, Beijing, China) according to the user manual. The slides were evaluated blindly by experienced pathologists, and histological scores were evaluated according to previous studies (33, 34) based on the following parameters: the area affected by inflammatory infiltration and tissue damage.

### Histological analysis and grades

Three random areas of each colon sample were photographed, with the grades of inflammatory cell infiltration and tissue damage scored according to the following criteria: (1) Inflammatory cell infiltration: 0 point, no inflammatory infiltration; 1 point, less inflammatory infiltration in the lamina propria; 2 points, the muscular layer of the mucosa infiltrates, thickening of the mucosa; 3 points, inflammatory cells penetrate the submucosa; (2) tissue damage: 0 point, no epithelial cell changes; 1 point, goblet cell deletion; 2 points, a large area of goblet cells is missing, crypt is missing; 3 points, extensive destruction of the structure of the mucosa and extension to the muscular layer of the intestine. The mean of scores of the three areas represented the grade of colitis in each animal.

### Immune fluorescence staining and measurement

Immunostainings on 4-μm paraffin sections were performed as described previously (35). The following primary antibodies were used: MMP3 antibody (Abcam, #AB52915, 1:100), MMP8 antibody (Abcam, #AB53017, 1:1,000), MMP10 antibody (Abcam, #AB261733, 1:100), IL6 antibody (CST, #12912s, 1:200), IL1A antibody (Abcam, #AB300499, 1:50), IL1B antibody (Abcam, #ab234437, 1:50), CXCL2 antibody (Thermo, #MA5-23737, 1:100), CXCL3 antibody (Abcam, #ab220431, 1:1,000), S100A8/S100A9 antibody (Abcam, #ab288715, 1:500), NF-κB p65 antibody (Abcam, #ab16502, 1:1,000), Phospho-NF-κB p65 antibody (CST, #3033, 1:1,000), TRAF6 antibody (Abcam, #ab33915, 1:1,000), and IRAK-1 antibody (Abcam, #ab218130, 1:200). Secondary antibodies were conjugated with AlexaFluor 488 (Thermo Fisher Scientific, Massachusetts, USA, 1:1,000). Nuclei were counterstained with DAPI (Sigma, #MBD0015, 1:10,000). Fluorescent images were captured with a confocal laser microscope (Spin SR10, Olympus, Japan) and processed with Adobe Photoshop software.

The fluorescence signal intensities of MMP3, MMP8, MMP10, IL6, IL1A, IL1B, CXCL2, CXCL3, S100A8+S100A9, NF-κB (p65), Phospho-NF-κB (p65), TRAF6 and IRAK1 were measured using ImageJ on three sections, which came from every ninth serial sections from distal colon of three WT mice and three miR-146a+/- mice; three DSS-treated WT mice and three DSS-treated miR-146a+/- mice in experiment 1, and every ninth serial sections from distal colon of three saline treated miR-146a-/- mice, three saline + DSS treated miR-146a-/- mice and four mimics + DSS miR-146a-/- mice in experiment 2, and three every ninth serial sections from distal colon of three saline treated WT mice, three saline + DSS treated WT mice and four mimics + DSS WT mice.

### Cy3-conjugated mimics treatment

Sixteen 8-week-old C57BL/6N mice were randomly divided into four groups and fasted for 6 h, and then mice were intraperitoneally injected or administrated via gavage with *miR-146a*-*5p* mimics (6.5 pg per gram of body weight) and *miR-146a*-*3p* mimics (3.25 pg per gram of body weight), or treated in combination with *miR-146a*-*5p* mimics (6.5 pg per gram of body weight) and *miR-146a*-*3p* mimics (3.25 pg per gram of body weight), or served as untreated controls. Colon tissues were collected 4 h after treatment of *miR-146a* mimics and fixed in 4% PFA overnight. Colon tissues were embedded in OCT after immersion in 15% sucrose and 30% sucrose overnight, and sectioned in a cryostar (NX50, Thermo Fisher Scientific) at a thickness of 14 μm. The colon cryosections were stained with DAPI and photographed using a confocal microscope (Zeiss 710, Germany).

## Results

### Haploinsufficiency of *miR-146a* augmented DSS-induced IBD

**Figure 1:**
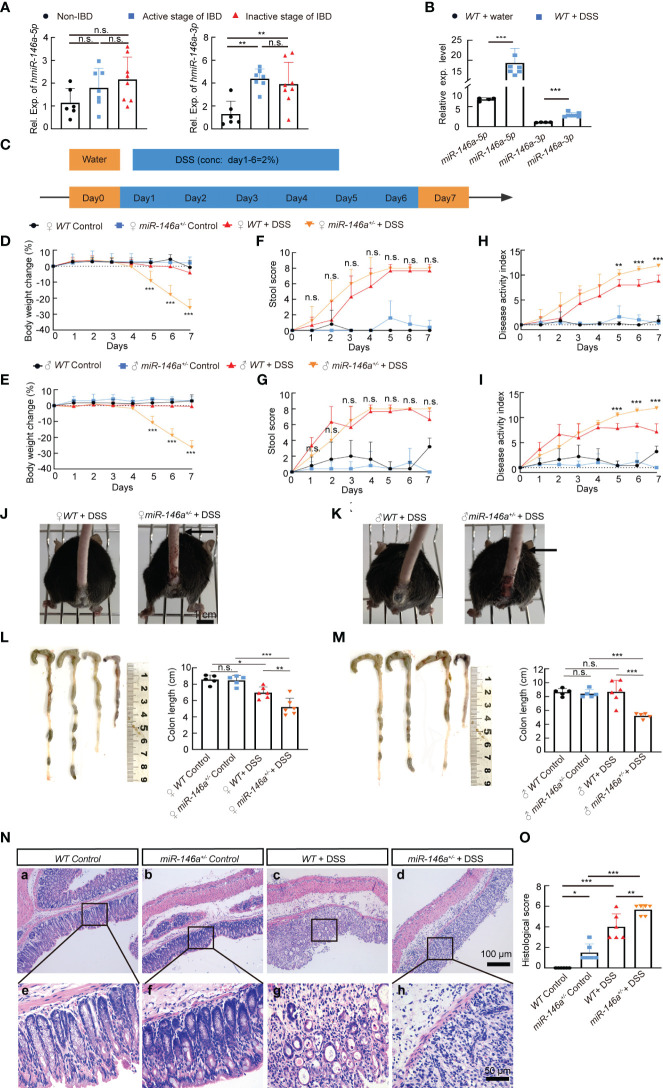
Haploinsufficiency of *miR-146a* led to severe inflammatory bowel disease (IBD). **(A)** The expression levels of *miR-146a*-5p and *miR-146a*-3p in the colon of patients without IBD (*n* = 6) or with IBD in the active stage (*n* = 7) or the inactive stage (*n* = 8) was quantified by RT-qPCR; the results represent two independent experiments with triplicates of each sample. **(B)** The expression level of *miR-146a*-5p and *miR-146a*-3p in the colon of *WT* mice treated with (*n* = 4) or without DSS (*n* = 7); the results represent two independent experiments with triplicates of each sample. **(C)** The schematic view of the experiment procedure of the DSS-induced IBD mouse model. **(D–I)** Body weight change **(D, E)**, stool score **(F, G)**, and disease activity index **(H, I)** of female [**(D, F, H)**, *n* = 5, 5, 6, and 8] and male [**(E, G, I)**, *n* = 5, 5, 6, and 8] *WT* and *miR-146a^+/-^* mice with or without 2% DSS treatment for 6 days. The results represent three independent animal experiments. **(J, K)** All 2% DSS-treated female [**(J)**, image on the right] and male [**(K)**, image on the right] *miR-146a^+/-^* mice had massive rectal bleeding (arrows point to the end of bloodstain on tails), but none of the 2% DSS-treated *WT* female [**(G)**, left] and male [**(H)**, left] mice did. **(L, M)** Colon length of female [**(L)**, *n* = 5, 5, 6, and 6], and male [**(M)**, *n* = 5, 5, 6, and 5] *WT* and *miR-146a^+/-^* mice treated with or without 2% DSS. Mean ± SD: female groups: *WT* Control = 8.56 ± 0.56 cm, *miR-146a^+/-^* Control = 8.46 ± 0.62 cm, *WT*+DSS = 6.967± 0.70 cm, *miR-146a^+/-^* +DSS = 5.2 ± 0.38 cm; male groups: *WT* Control = 8.64 ± 0.52 cm, *miR-146a^+/-^* Control = 8.42 ± 0.58 cm, WT+DSS = 8.7 ± 1.64 cm, *miR-146a^+/-^* DSS = 5.22 ± 0.38 cm. **(N, O)** Hematoxylin and eosin (HE) staining of the sagittal section of the colon derived from *WT* and *miR-146a^+/-^* mice treated with or without 2% DSS **(N)** and histological score of HE-stained sections **(O)**, *n* = 6. All data were shown as mean ± SD *p < 0.05, **p < 0.01, ***p < 0.001, Student’s *t-*test was used in **(A, B)**, two-way ANOVA analysis was performed in **(D–I)**, and one-way ANOVA was applied in **(L–O)**. n.s., no significance.

To investigate whether *miR-146a* is involved in IBD, we employed RT-qPCR to quantify the expression level of *miR-146a* in the colon of patients, and the results demonstrated that *miR-146a-3p* was significantly expressed higher in the colon of patients with IBD in both the active stage and the inactive stage than that in non-IBD patients who had other types of bowel disease, and that there was an increasing trend in expression level of *miR-146a-5p* in the colon of patients with IBD compared to patients without IBD (**Figure 1A**). Since the expression of *miR-146a* could be increased in patients with other types of bowel diseases (36) and it is difficult to obtain colon tissues from healthy people, we generated an IBD mouse model by induction with 2.5% DSS and found that both *miR-146a-5p* and *miR-146a-3p* were upregulated by more than two fold in the colon of DSS-treated *wild type* (*WT*) mice when compared to untreated control *WT* mice (**Figure 1B**). These findings suggest that *miR-146a-5p* and *miR-146a-3p* were involved in the development of IBD.

To further decipher the function of *miR-146a* in IBD, we generated a global *miR-146a* knock-out (KO) mouse line using CRISPR/Cas9 technology (**Supplementary Figures S1A–C**) and used DSS to induce mice for the development of colitis symptoms (**Figure 1C**). During the treatment of 2% DSS in drinking water from day 1 to day 6, the body weight of DSS-treated female (**Figure 1D**) and male (**Figure 1E**) *miR-146a* ^+/-^ mice dropped significantly from day 5 to day 7 compared with DSS-treated *WT* mice, untreated control *miR-146a* ^+/-^, and *WT* mice, while DSS-treated female and male *WT* mice did not show significant body weight loss when compared to untreated *WT* control mice (**Figures 1D, E**). The stool scores of both DSS-treated *WT* and *miR-146a^+/-^* mice were dramatically increased from day 3 when compared to untreated controls, but there was no significant difference in stool score between DSS-treated *WT* and *miR-146a^+/-^* mice (**Figures 1F, G**). The DAI was significantly higher in DSS-treated female and male *miR-146a^+/-^* mice than in DSS-treated female and male *WT* mice (**Figures 1H, I**) from day 5 to day 7. Moreover, all 2% DSS-treated female and male *miR-146a^+/-^* mice had bloodstain on their tails, which indicated massive rectal bleeding, while 2% DSS-treated *WT* female and male mice did not (**Figures 1J, K**). Colon length and histological score are two key parameters that indicate the severity of colitis. The results showed that there was no difference in colon length between untreated *WT* and *miR-146a^+/-^* mice, and that 2% DSS treatment significantly shortened colon length (19%) in *WT* female, but not male, mice and the colon lengths of DSS-treated female and male *miR-146a^+/-^* mice were shortened by 39% and 38% when compared to untreated female and male *miR-146a^+/-^* control mice, and were also significantly shorter than DSS-treated female and male *WT* mice, respectively (**Figures 1L, M**). Colon sections of different groups were histologically stained and examined. We found that DSS treatment caused significant destruction of epithelial layer and induced inflammation in the colon of *WT* mice when compared to untreated *WT* control, and the colon derived from DSS-treated *miR-146a^+/-^* mice showed a higher histological score, with more inflammatory cell infiltration and tissue damage, than untreated *miR-146a^+/-^* and DSS-treated *WT* mice. Interestingly, untreated *miR-146a^+/-^* mice exhibited a slightly higher histological score, which can be attributed to inflammation when compared to untreated *WT* mice, indicating that *miR-146a* represses inflammation responses under physiological conditions (**Figures 1N, O**). These findings indicate that the insufficiency of *miR-146a* promotes the development of IBD from three aspects: colitis, bleeding, and body weight loss, and suggest that *miR-146a* plays a vital role in preventing the development of IBD.

### *MiR-146a* repressed multiple genetic regulatory networks in IBD

**Figure 2:**
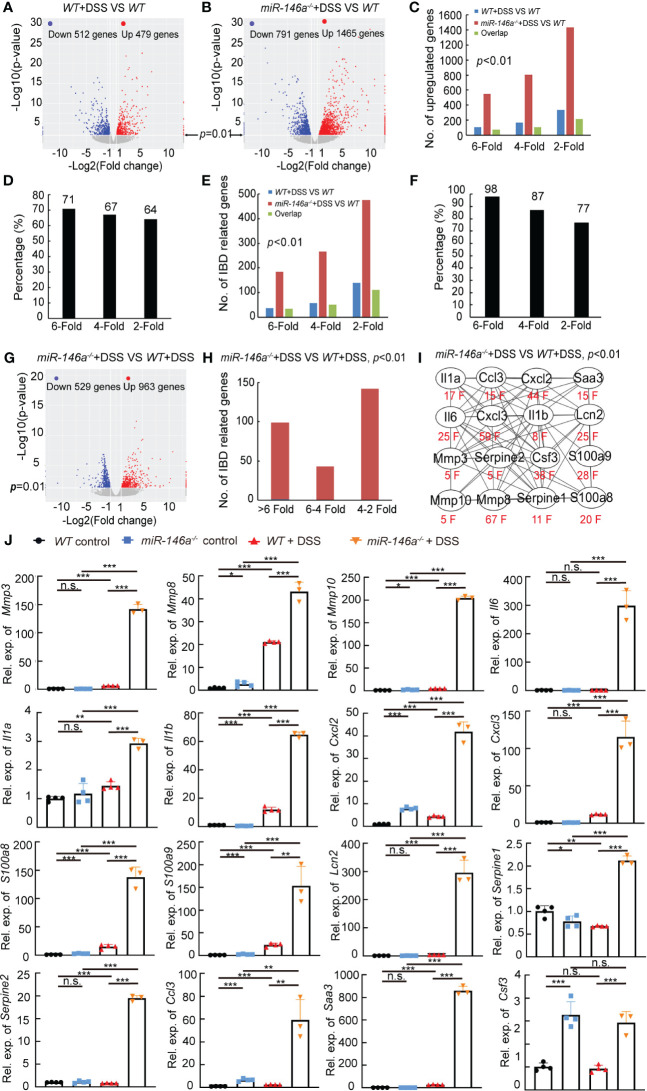
*MiR-146a* repressed DSS-induced IBD-related genes. **(A, B)** Volcano map of gene expressions (*p* < 0.01, |log2(Fold change)| > 1) in colons of DSS-treated *WT* **(A)** or *miR-146a^-/-^* mice **(B)** when compared to untreated *WT* mice. **(C)** Shared regulated genes in colons increased between DSS-treated *WT* and *miR-146a^-/-^* mice with different inclusion criteria. **(D)** Similar percentage of shared regulated genes in the colon between DSS-treated *WT* and *miR-146a^-/-^* mice with different inclusion criteria. **(E)** Number of shared regulated IBD-related genes in colons increased between DSS-treated *WT* and *miR-146a^-/-^* mice with different inclusion criteria. **(F)** Comparable percentage of shared regulated IBD-related genes in colons between DSS-treated *WT* and *miR-146a^-/-^* mice with different inclusion criteria. **(G)** Volcano plot of differentially expressed genes in colons of DSS-treated *miR-146a* ^-/-^ mice when compared to DSS-treated *WT* mice. **(H)** The number of upregulated IBD-related genes in colons of *miR-146a* ^-/-^ mice when compared to DSS-treated *WT* mice. **(I)** The fold change and the interaction network of selected representative upregulated IBD-related genes, *Il1a*, *Il1b*, *Il6*, *Ccl3*, *Cxcl2*, *Cxcl3*, *Saa3*, *Csf3*, *Lcn2*, *Mmp3*, *Mmp8*, *Mmp10*, *Serpine1*, *Serpine2*, *S100a8*, and *S100a9*. String APP in Cytoscape was used to analyze the protein functional network (37, 38). **(J)** The expression levels of *Mmp3*, *Mmp8*, *Mmp10*, *Il6*, *Il1a*, *Il1b*, *Cxcl2*, *Cxcl3*, *S100a8*, *S100a9*, *Lcn2*, *Serpine1*, *Serpine2*, *Ccl3*, *Saa3*, and *Csf3* in colons of *WT* control, DSS-treated *WT*, *miR-146a^-/^*,*^-^* and DSS-treated *miR-146a^-/-^* mice measured by RT-qPCR; the results represent two independent experiments with triplicates of each sample. Data were shown as mean ± SD *p < 0.05, **p < 0.01, ***p < 0.001, Student’s *t*-*test*, *n* = 4, 4, and 3. n.s., no significance.

We next investigated the genetic regulatory network regulated by *miR-146a* in IBD. Total RNA was extracted from colons of three untreated *WT* mice, three 2% DSS-treated *WT* mice, and three DSS-treated *miR-146a^-/-^* mice. RNA sequencing data demonstrated that 479 and 512 genes that met the criteria of *p* < 0.01 and |log2(Fold change)| > 1 were up- and downregulated in *WT* mice after DSS treatment, respectively (**Figure 2A**; **Supplementary Table S4**). However, much more genes (1,465 versus 479) were upregulated and 791 genes were downregulated in DSS-treated *miR-146a^-/-^* mice with the same criteria compared to untreated *WT* mice (**Figure 2B**; **Supplementary Table S4**). Maintaining *p* < 0.01 but lowering fold-change criteria from sixfold to fourfold or to twofold led to a disproportionate increase in upregulated genes shared between DSS-treated *WT* and *miR-146a^-/-^* mice and maintaining close to 67% shared gene regardless of fold-change threshold (**Figures 2C, D**). A disproportionate increase in IBD-related genes was also observed to be shared between DSS-treated *WT* and *miR-146a^-/-^* mice when lowering the fold-change criteria and maintaining more than 77% shared IBD-related gene from change thresholds of twofold (77%) to sixfold (98%) (**Figures 2E, F**). When increasing fold-change threshold to 10, 28 out of 71 upregulated genes in DSS-treated *WT* mice were IBD-related genes, while 128 out of 381 upregulated genes in DSS-treated *miR-146a^-/-^* mice were IBD-related genes, and 29 (such as *Il1a*, *Il11*, *Il5ra*, *Il1r2*, *Cxcl13*, *Cxcl5*, *Osm*, *Arg1*, *Igfbp5*, and *Vsig4*) out of 128 genes were predicted to be direct target genes of *miR-146a* by TargetScan8.0 (**Supplementary Figures S2A, B**; **Supplementary Table S5**). When further elevating fold-change threshold to 45, the number of upregulated IBD-related genes in DSS-treated *miR-146a^-/-^* mice was 10 times more (55 versus 5) than that in DSS-treated *WT* mice (**Supplementary Figures S2C, D**). However, the number of genes downregulated more than 10-fold in DSS-treated *miR-146a^-/-^* mice was not remarkably increased when compared to DSS-treated *WT* mice (64 versus 45, **Supplementary Figures S2E, F**; **Supplementary Table S5**). These findings suggest that *miR-146a* mainly inhibits expression of DSS-induced genes, especially IBD-related genes.

Further analyses found that 963 and 529 genes that met the criteria of *p* < 0.01 and |log2(fold change)| > 1 were up- and downregulated in DSS-treated *miR-146a^-/-^* when compared to DSS-treated *WT* mice, respectively (**Figure 2G**; **Supplementary Figure S3A**). Principal component analysis also showed that the mRNA expression profile in the colon of DSS-treated *miR-146a^-/-^* mice was separated from DSS-treated *WT* mice and naïve mice (**Supplementary Figure S3B**).When increasing fold-change threshold to 10, 225 and 41 genes were up- and downregulated in DSS-treated *miR-146a^-/-^* mice when compared to DSS-treated *WT* mice, respectively, and 64 out of 225 were IBD-related genes (**Supplementary Figures S3C, D**). Hundreds of IBD-related genes were further induced by DSS in *miR-146a^-/-^* mice more than twofold and 141 IBD-related genes were upregulated by more than fourfold when compared to DSS-treated *WT* mice (**Figure 2H**; **Supplementary Figure S3E**). These upregulated IBD-related genes belong to genetic regulatory networks of innate immune responses, intestinal epithelial and vascular endothelial barriers, and coagulation, corresponding to all symptoms of IBD (colitis, bleeding, bowel problems, and body weight loss) (**Supplementary Figures S3C, E**). It is known that cytokines, chemokines, and other pro-inflammatory factors are upregulated in patients with IBD (39–41). Matrix metalloproteinases (MMPs) destroy the extracellular matrix, damage the intestinal epithelial barrier and vascular endothelial barrier, and subsequently affect digestion and absorption, leading to bleeding (6). Another reason for massive bleeding is dysfunction in coagulation (13). Thus, 16 out of 141 further upregulated more than fourfold genes, which shows that multiple genetic regulatory networks involved in IBD were selected for validation: *Il1a*, *Il1b*, and *Il6* from the cytokine family; *Ccl3*, *Cxcl2*, and *Cxcl3* from the chemokine family; Serum Amyloid A3 (*Saa3*), colony stimulating factor 3 (*Csf3*), Lipocalin-2 (*Lcn2*), *S100a8*, and *S100a9* from pro-inflammatory factors; *Mmp3*, *Mmp8*, and *Mmp10* from the MMP family; and *Serpine1* and *Serpine2* from the Serpin family, which inhibits serine proteases including thrombin, urokinase, and plasmin and is related to coagulation (**Figure 2I**; **Table 1**).

**Table 1:** Expression of 16 genes in *WT*, DSS-treated *WT*, and *miR-146a^-/-^* mice.

Gene	*WT*	*WT* + DSS	*miR-146a^-/-^* + DSS	*WT* + DSS/WT	*miR-146a^-/-^* + DSS/WT + DSS
*Mmp3*	0.84	10.09	50.07	12.05	4.96
*Mmp8*	0.01	0.37	25.39	36.05	68.16
*Mmp10*	0.75	11.06	54.15	14.79	4.90
*Il6*	0.03	0.09	2.19	3.17	24.60
*Il1α*	0.21	0.97	16.31	4.59	16.82
*Il1β*	0.76	8.96	75.06	11.77	8.38
*Cxcl2*	0.02	0.57	24.69	24.66	43.51
*Cxcl3*	0.01	0.21	12.35	18.53	59.45
*S100a8*	0.18	7.71	155.48	43.08	20.17
*S100a9*	0.14	11.72	329.84	85.17	28.14
*Lcn2*	0.24	1.46	36.36	6.11	24.95
*Serpine1*	0.37	0.99	10.79	2.69	10.85
*Serpine2*	0.85	1.92	9.58	2.27	5.00
*Ccl3*	0.62	1.94	28.83	3.15	14.83
*Saa3*	0.77	68.38	1025.04	89.37	14.99
*Csf3*	0.03	0.37	14.38	13.73	38.42

**Figure 3:**
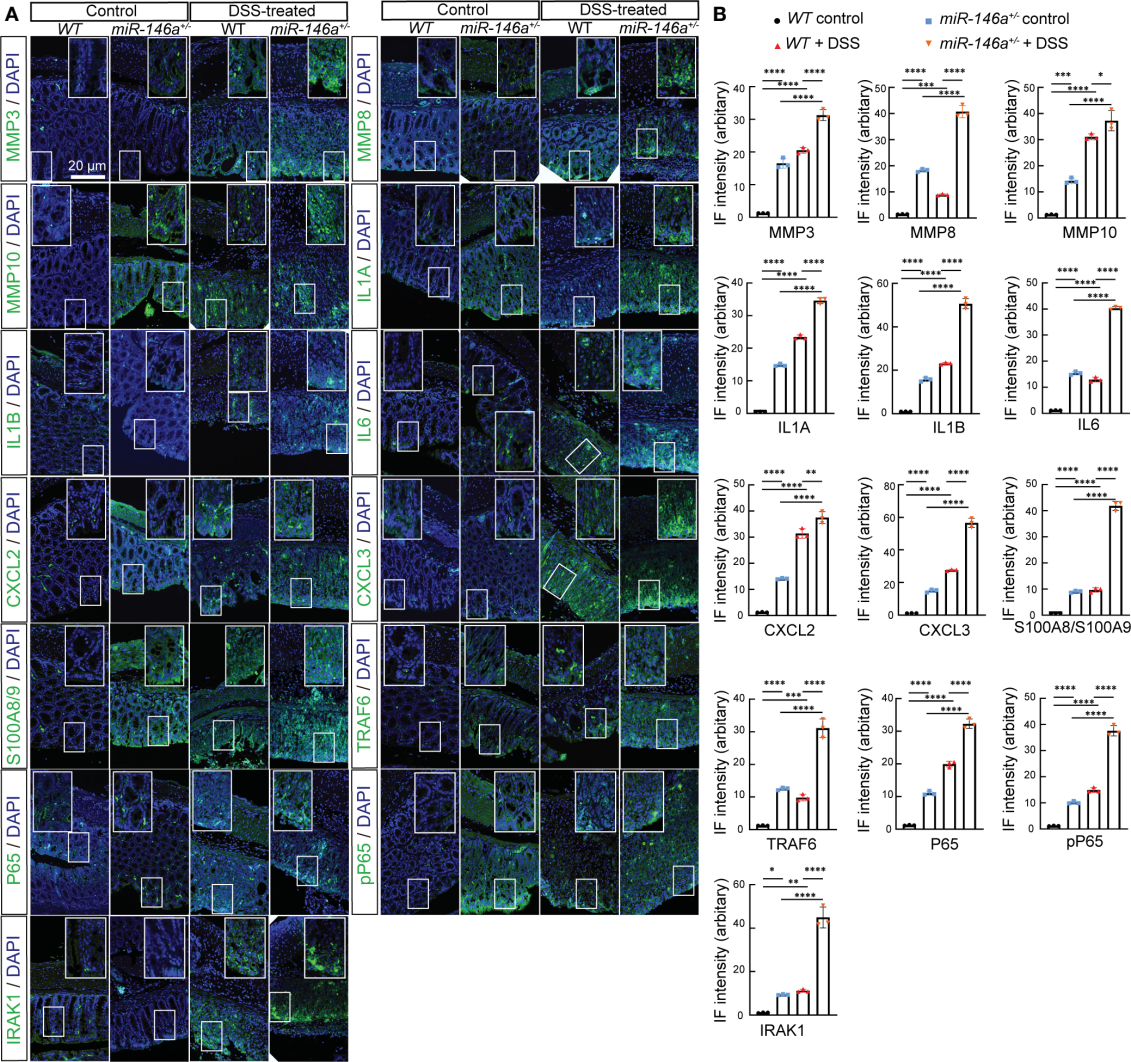
Haploinsufficiency of *miR-146a* led to further upregulation of DSS-induced IBD-related genes. **(A)** Double staining with DAPI (blue) and antibody (green) against MMP3, MMP8, MMP10, IL1A, IL1B, IL6, CXCL2, CXCL3, S100A8/A9, TRAF6, P65, pP65, or IRAK1 on colon sections derived from control *WT*, DSS-treated *WT*, and DSS-untreated and -treated *miR-146a^+/-^ mice.* The inset images are the high-magnification view of the boxed area on the bottom of the corresponding images. **(B)** Quantification of immune fluorescence intensity. Data were shown as mean ± SD *p < 0.05, **p < 0.01, ***p < 0.001, ****p < 0.001, one-way ANOVA, *n* = 3. The results represent three independent staining experiments.

Quantitative RT-PCR confirmed the dramatic upregulation of these 16 genes in the colon of DSS-treated *miR-146a^-/-^* mice compared to DSS-treated *WT* mice, and the expression levels of *Mmp3*, *Mmp8*, *Mmp10*, *Il1a*, *Il1b*, *Cxcl2*, *Cxcl3*, *S100a8*, *S100a9*, *Lcn2*, *Ccl3*, and *Saa3* increased in the colon of DSS-treated *WT* mice compared to *WT* control mice (**Figure 2J**). It is also interesting to find that the expression levels of *Mmp8*, *Mmp10*, *Cxcl2*, *Cxcl3*, *S100a8*, *S100a9*, *Ccl3*, and *Csf3* increased in the colon of DSS-treated *WT* mice comparing to *WT* control mice (**Figure 2J**). Further immunostaining demonstrated that the protein expression levels of MMP3, MMP8, MMP10, IL1A, IL1B, IL6, CXCL2, CXCL3, S100A8, and S100A9 significantly increased in the colon of *WT* mice after DSS treatment, and further increased in the colon of DSS-treated *miR-146a^+/-^* mice. Since it was reported that *miR-146a* negatively regulated TRAF6 and IRAK1, and that p65 (RelA) is a predicted target of *miR-146a*-3p, we examined and found that the expression of TRAF6, IRAK1, p65, and p-p65 (phosphorylated p65) was also further upregulated in the colon of DSS-treated *miR-146a^+/-^* mice when compared to DSS-treated *WT* mice (**Figures 3A, B**). Note that the expression levels of MMP3, MMP8, MMP10, IL1A, IL1B, IL6, CXCL2, CXCL3, S100A8, S100A9, TRAF6, P65, pP65, and IRAK1 increased in the colon of untreated *miR-146a^+/-^* mice when compared to untreated *WT* mice, indicating that *miR-146a* prevents the allergic response of the intestine to a normal environment (**Figures 3A, B**). These findings suggest that de-repression of genetic regulatory networks of immunity, intestinal epithelial and vascular endothelial barriers, and coagulation is the molecular mechanism for massive bleeding and body weight loss in DSS-treated *miR-146a* KO mice.

### Supplementation with *miR-146a mimics* attenuated DSS-induced IBD in *miR-146a^-/-^* mice

**Figure 4:**
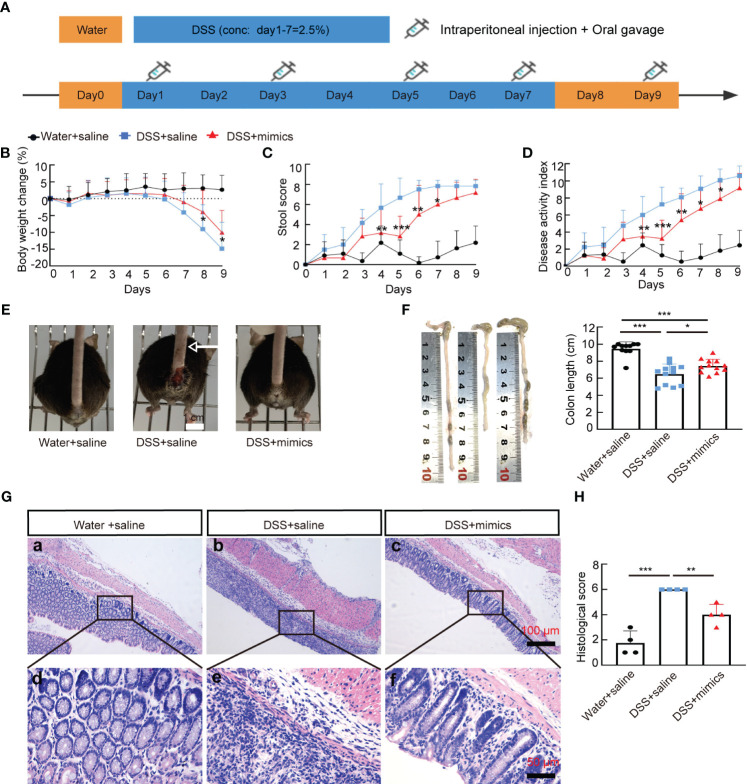
*MiR-146a* mimics alleviated DSS-induced IBD in *miR-146a^-/-^* mice. **(A)** The scheme of the rescue experiment. **(B–D)** Body weight change **(B)**, stool score **(C)**, and disease activity index **(D)** of saline-treated (*n* = 11), DSS + saline-treated (*n* = 12), and DSS + *miR-146a* mimics-treated (*n* = 12) *miR-146a^-/-^* mice. The results represent three independent animal experiments. **(E)** All 2% DSS + saline-treated *miR-146a^-/-^* mice (middle) had severe rectal bleeding (arrows point to the end of bloodstain on tails), but neither of the saline-treated nor DSS + *miR-146a* mimics-treated *miR-146a^-/-^* mice did. **(F)** Colon length of saline-treated (*n* = 11), DSS + saline-treated (*n* = 12), and DSS + *miR-146a* mimics-treated (*n* = 12) *miR-146a^-/-^* mice. Mean ± SD: Water + saline = 9.455 ± 0.81 cm, DSS + saline = 6.475 ± 1.2 cm, and DSS + mimics = 7.442 ± 0.79 cm. **(G, H)** HE staining of the sagittal section of colons derived from saline-treated, DSS + saline-treated, and DSS + *miR-146a* mimics-treated *miR-146a^-/-^* mice **(G)** and histological score of HE-stained sections **(H)**, *n* = 4. All data were shown as mean ± SD *p < 0.05, **p < 0.01, ***p < 0.001, two-way ANOVA was used in **(B–D)** and one-way ANOVA was used in **(F–H)**.

Next, we examined whether supplementation with *miR-146a* mimics could relieve DSS-induced IBD in *miR-146a^-/-^* mice. *MiR-146a^-/-^* mice were divided into three groups, treatment with saline, treatment with 2% DSS and saline, treatment with 2% DSS and *miR-146a* mimics (**Figure 4A**). The results showed that 2% DSS induced severe IBD in *miR-146a^-/-^* mice with obvious body weight loss and an increase in stool score and DAI, and that the combination of gavage administration and intraperitoneal injection of the modified *miR-146a-5p* and *miR-146a-3p* mimics for four times every other day significantly reduced body weight loss, stool score, and DAI in DSS-treated *miR-146a^-/-^* mice when compared to DSS + saline-treated *miR-146a^-/-^* mice (**Figures 4B–D**). DSS-induced spouted bleeding of *miR-146a^-/-^* mice was eliminated by supplementation with *miR-146a* mimics (**Figure 4E**). *MiR-146a* mimics prevented 32% DSS-induced reduction in the colon length and attenuated the destruction of the intestine as well as inflammatory cell filtration (**Figures 4F–H**). These results demonstrated that supplementation with *miR-146a* mimics can relieve DSS-induced IBD in *miR-146a^-/-^* mice.

### *MiR-146a mimics* represses expression of multiple genetic regulatory networks

**Figure 5:**
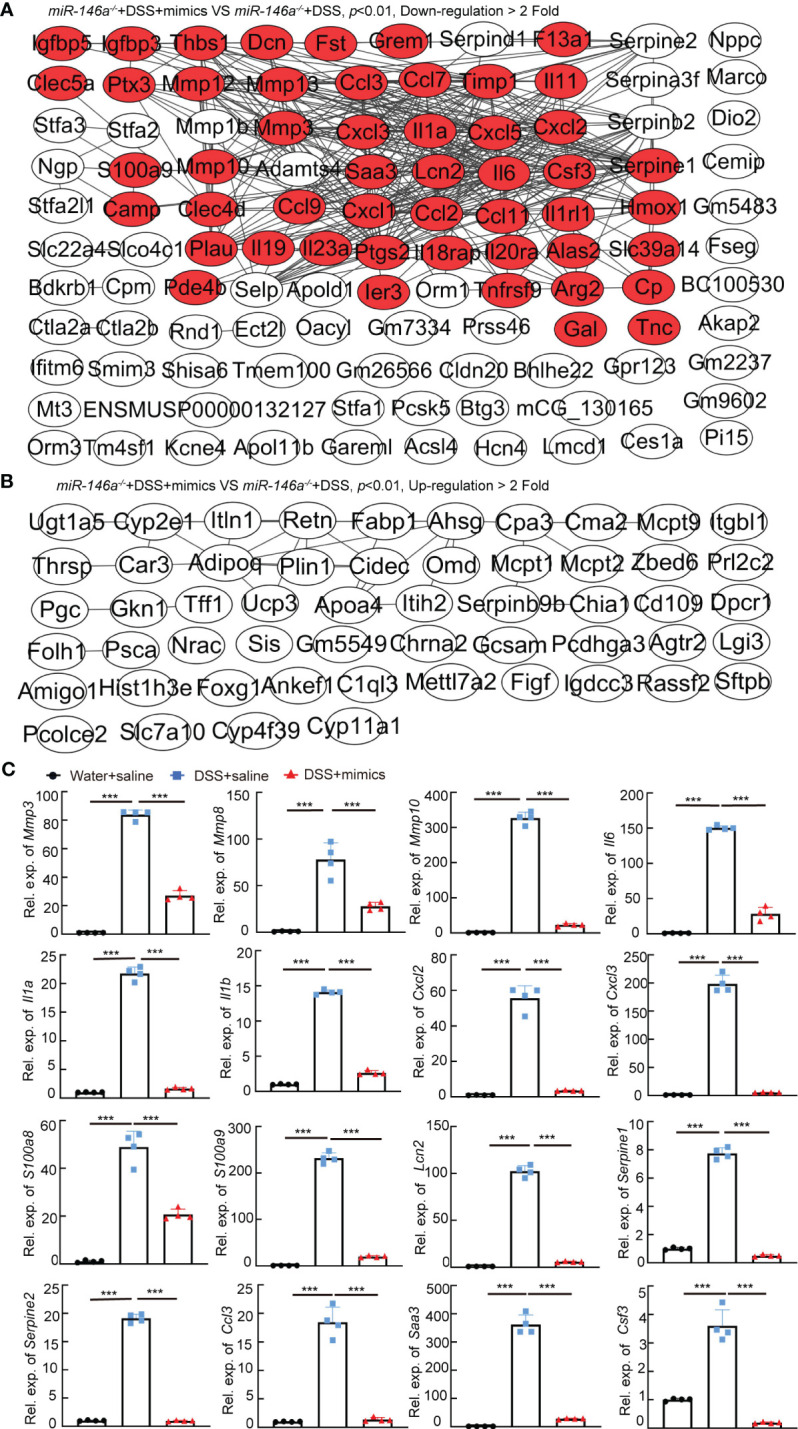
The regulatory effects of *miR-146a* mimics on DSS-induced gene regulatory network in colons of *miR-146a^-/-^* mice. **(A)** The functional interaction network of downregulated genes by *miR-146a* mimics in the colon of DSS-treated *miR-146a^-/-^* mice was analyzed using String database APP in Cytoscape. Out of the 135 downregulated genes, 108 with or without predicted connections (gray lines, inclusion criteria: downregulated fold **≥** 2 and *p* value <0.01) were shown. Red marked IBD-related genes. Data were derived from five *miR-146a* mimics + DSS-treated *miR-146a^-/-^* mice and six saline + DSS-treated *miR-146a^-/-^* mice. **(B)** The functional interaction network of upregulated genes by *miR-146a* mimics in colons of DSS-treated *miR-146a^-/-^* mice was analyzed using String database APP in Cytoscape. Out of the 69 upregulated genes, 54 with or without predicted connections (gray lines, inclusion criteria: upregulated fold **≥** 2 and *p* value <0.01) were shown. **(C)** The expression levels of *Mmp3*, *Mmp8*, *Mmp10*, *Il6*, *Il1a*, *Il1b*, *Cxcl2*, *Cxcl3*, *S100a8*, *S100a9*, *Lcn2*, *Serpine1*, *Serpine2*, *Ccl3*, *Saa3*, and *Csf3* in colons of saline-treated, DSS + saline-treated, and DSS + *miR-146a* mimics-treated *miR-146a^-/-^* mice measured by RT-qPCR; the results represent two independent experiments with triplicates of each sample. Data were shown as mean ± SD ***p < 0.001, one-way ANOVA, *n* = 4, 4, and 4.

**Figure 6:**
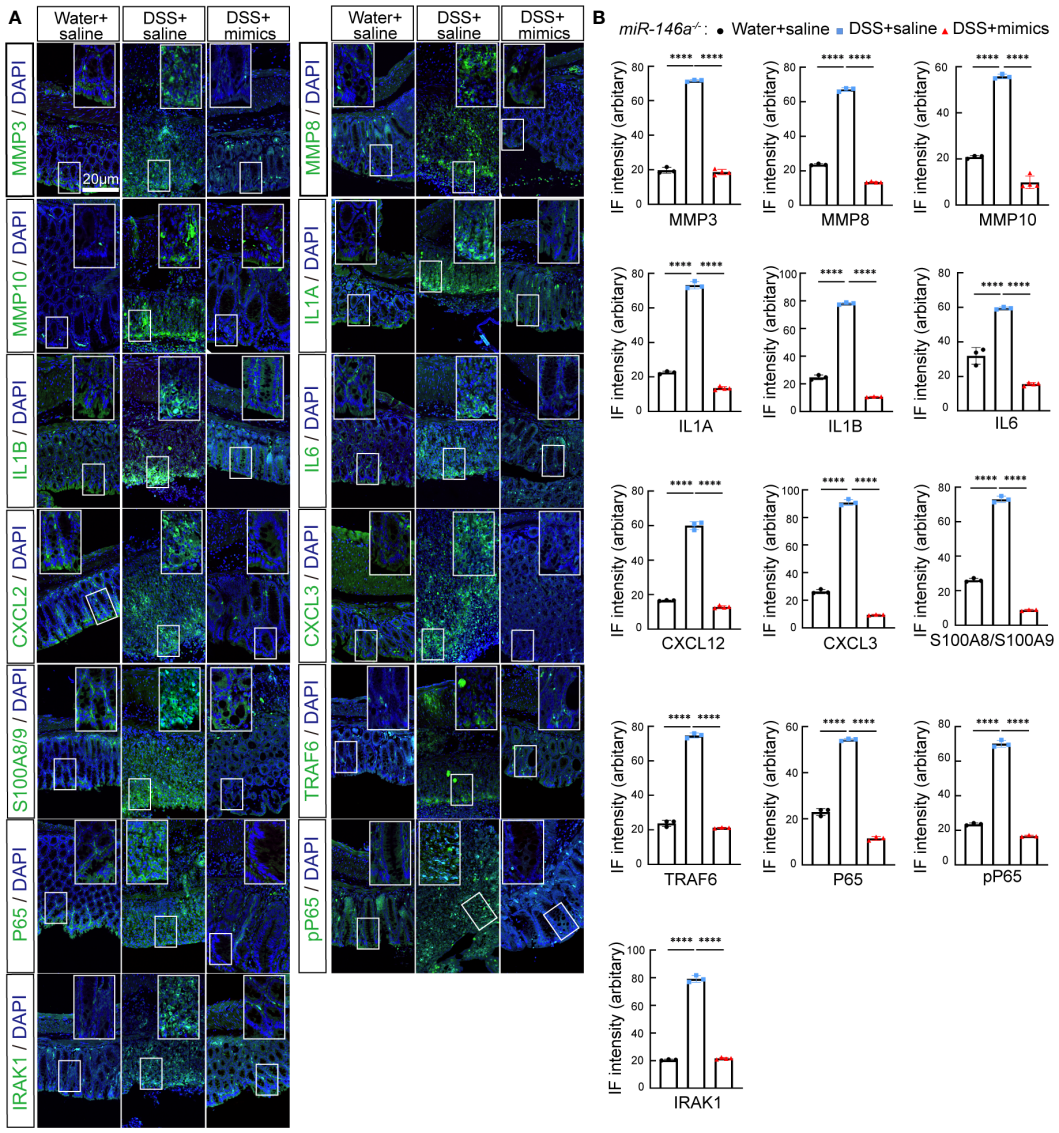
*MiR-146a* mimics repressed DSS-induced IBD-related genes in colons of *miR-146a^-/-^* mice. **(A)** Double staining with DAPI (blue) and antibody (green) against MMP3, MMP8, MMP10, IL1A, IL1B, IL6, CXCL2, CXCL3, S100A8/A9, TRAF6, P65, pP65, or IRAK1 on colon sections derived from saline-treated, DSS + saline-treated, and DSS + *miR-146a* mimics-treated *miR-146a^-/-^* mice. The inset images are the high-magnification view of the boxed area on the bottom of the corresponding images. **(B)** Quantification of immune fluorescence intensity. Data were shown as mean ± SD ****p < 0.001, one-way ANOVA, (*n* = 3, 3, and 4). The results represent three independent staining experiments.

To identify the downregulated IBD-related genes by *miR-146a* mimics, RNA sequencing was performed and the results illustrated that 135 and 69 genes that met the criteria of *p* < 0.01 and |log2(Fold change)| > 1 were down- and upregulated in the colon of *miR-146a* mimics + DSS-treated *miR-146a^-/-^* mice, when compared to DSS + saline-treated *miR-146a^-/-^* mice, respectively, and 50 out of the 135 genes were downregulated IBD-related genes (**Figures 5A, B**; **Supplementary Table S6**). Principal component analysis showed that the mimics treatment pulled the expression profile back towards health status (**Supplementary Figure S4**). Quantitative RT-PCR showed that the expression levels of *Mmp3*, *Mmp8*, *Mmp10*, *Il1a*, *Il6*, *Il1b*, *Cxcl2*, *Cxcl3*, *S100a8*, *S100a9*, *Lcn2*, *Serpine1*, *Serpine2*, *Ccl3*, *Saa3*, and *Csf3* were induced in the colon of DSS + saline-treated *miR-146a^-/-^* mice, but reversed by *miR-146a* mimics (**Figure 5C**). Further immunostaining demonstrated that the expression levels of MMP3, MMP8, MMP10, IL1A, IL1B, IL6, CXCL2, CXCL3, S100A8, S100A9, TRAF6, p65, p-p65, and IRAK1 were induced by DSS in the colon of *miR-146a^-/-^* mice, but repressed by *miR-146a* mimics (**Figures 6A, B**).

### *MiR-146a mimics* relieved DSS-induced colitis in *WT* mice

**Figure 7:**
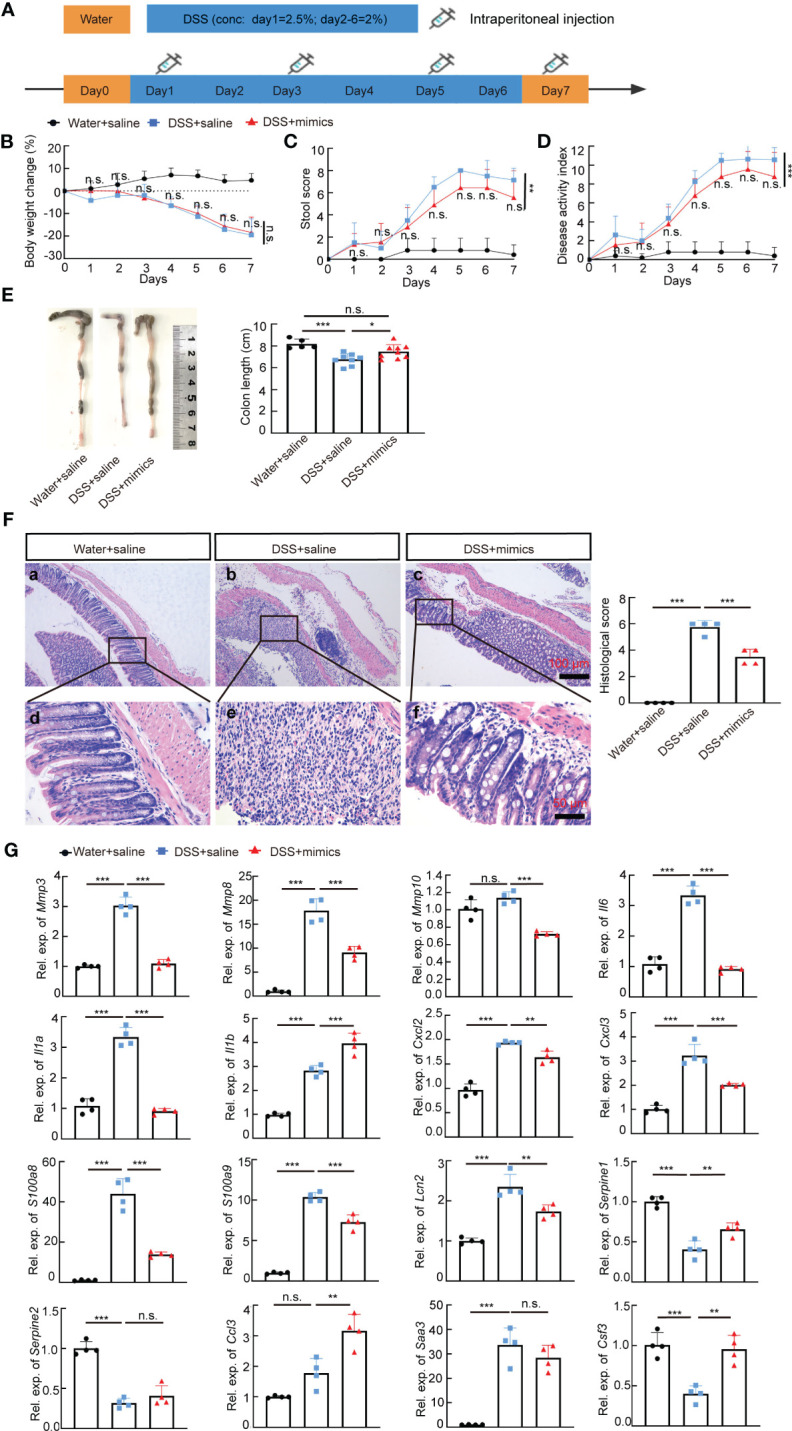
M*iR-146a* mimics relieved IBD symptoms through regulating DSS-induced IBD-related genes in *WT* mice. **(A)** The scheme of the experiment procedure. **(B–D)** Body weight change **(B)**, stool score **(C)**, and disease activity index **(D)** of saline-treated, DSS + saline-treated, and DSS + *miR-146a* mimics-treated *WT* mice, *n* = 5, 8, and 9. **(E)** Colon length of saline-treated, DSS + saline-treated, and DSS+*miR-146a* mimics-treated *WT* mice, *n* = 5, 8, and 9. Mean ± SD: Water + saline = 8.18 ± 0.44 cm, DSS + saline = 6.75 ± 0.54 cm, and DSS + mimics = 7.467 ± 0.644 cm. The results represent three independent animal experiments. **(F)** HE staining of sagittal sections of colon derived from saline-treated, DSS + saline-treated, and DSS + *miR-146a* mimics-treated *WT* mice and histological score of HE-stained sections, *n* = 4. **(G)** The expression levels of *Mmp3*, *Mmp8*, *Mmp10*, *Il6*, *Il1a*, *Il1b*, *Cxcl2*, *Cxcl3*, *S100a8*, *S100a9*, *Lcn2*, *Serpine1*, *Serpine2*, *Ccl3*, *Saa3*, and *Csf3* in colons of saline-treated, DSS + saline-treated, and DSS + *miR-146a* mimics-treated *WT* mice measured by RT-qPCR. *n* = 4, 4, and 4. All data were shown as mean ± SD *p < 0.05, **p < 0.01, ***p < 0.001, two-way ANOVA was used in **(B–D)** and one-way ANOVA was used in **(E–G)**. n.s., no significance.

**Figure 8:**
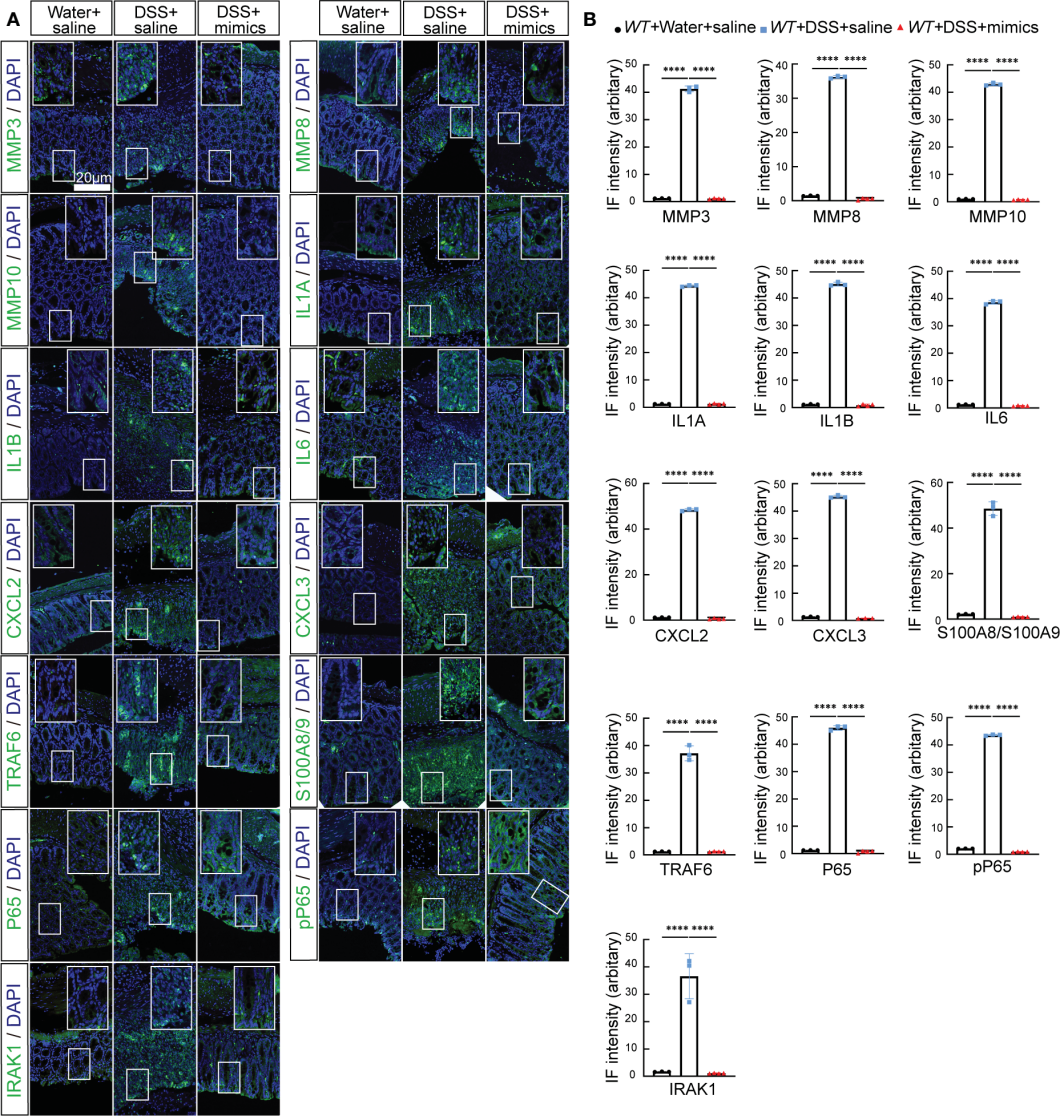
*MiR-146a* mimics repressed DSS-induced IBD-related genes in colons of WT mice. **(A)** Double staining with DAPI (blue) and antibody (green) against MMP3, MMP8, MMP10, IL1A, IL1B, IL6, CXCL2, CXCL3, S100A8/A9, TRAF6, P65, pP65, or IRAK1 on colon sections derived from saline-treated, DSS + saline-treated, and DSS + miR-146a mimics-treated WT mice. The inset images are the high-magnification view of the boxed area on the bottom of the corresponding images. **(B)** Quantification of immune fluorescence intensity. Data were shown as mean ± SD, ****p < 0.0001, one-way ANOVA, *n* = 3, 3, and 4. The results represent three independent staining experiments.

We next examined whether *miR-146a* mimics could attenuate DSS-induced IBD in *WT* mice. *WT* mice were divided into three groups. Two groups of mice were fed with 2.5% DSS in water on day 1, followed by 2% DSS from day 2 to day 6, and water on day 7; the other group of mice were fed with water (**Figure 7A**). The results showed that DSS induced severe IBD in *WT* mice with obvious body weight loss and an increase in stool score and DAI, and that intraperitoneal injection of *miR-146a-5p* and *miR-146a-3p* mimics for four times every other day did not reduce body weight loss, but decreased stool score and DAI in DSS-treated *WT* mice when compared to DSS + saline-treated *WT* mice (**Figures 7B–D**). Moreover, *miR-146a* mimics prevented DSS-induced reduction in colon length of *WT* mice (**Figure 7E**), and decreased damage to the intestine as well as inflammatory cell filtration (**Figure 7F**). Quantitative RT-PCR showed that the expression levels of *Mmp3*, *Mmp8*, *Mmp10*, *Il6*, *Il1a*, *Cxcl2*, *Cxcl3*, *S100a8*, *S100a9*, and *Lcn2* were induced in the colon of DSS + saline-treated *WT* mice, but significantly downregulated by *miR-146a* mimics (**Figure 7G**). The upregulation of *Saa3* was not significantly repressed by *miR-146a* mimics, and downregulation of *Serpine1*, *Serpine2*, and *Csf3* was detected in DSS + saline-treated *WT* mice when compared to saline-treated *WT* mice. *MiR-146a* mimics restored the expression levels of *Serpine1* and *Csf3* (**Figure 7G**). Surprisingly, the expression of *Il1b* and *Ccl3* was further increased in the colon of *miR-146a* mimics + DSS-treated *WT* mice compared to DSS + saline-treated *WT* mice. Further immunostaining illustrated that the expression levels of MMP3, MMP8, MMP10, IL1A, IL1B, IL6, CXCL2, CXCL3, S100A8, S100A9, TRAF6, p65, p-p65, and IRAK1 were induced by DSS in the colon of *WT* mice, but reversed by *miR-146a* mimics (**Figure 8**). To investigate whether the modified *miR-146a* mimics were uptaken by intestinal cells, Cy3-conjugated *miR-146a*-5p mimics and Cy3-conjugated *miR-146a-3p* mimics were administrated into *WT* mice via gavage or intraperitoneal injection or combinatory injection, and the fluorescence images captured by confocal microscopy showed that *miR-146a* mimics were uptaken by intestinal cells 4 h after administration, and more absorption of *miR-146a* mimics was found in the gavage and intraperitoneal injection combination group (**Supplementary Figure S5**).

### The expression level of IBD-related genes in colon samples from patients with IBD

**Figure 9:**
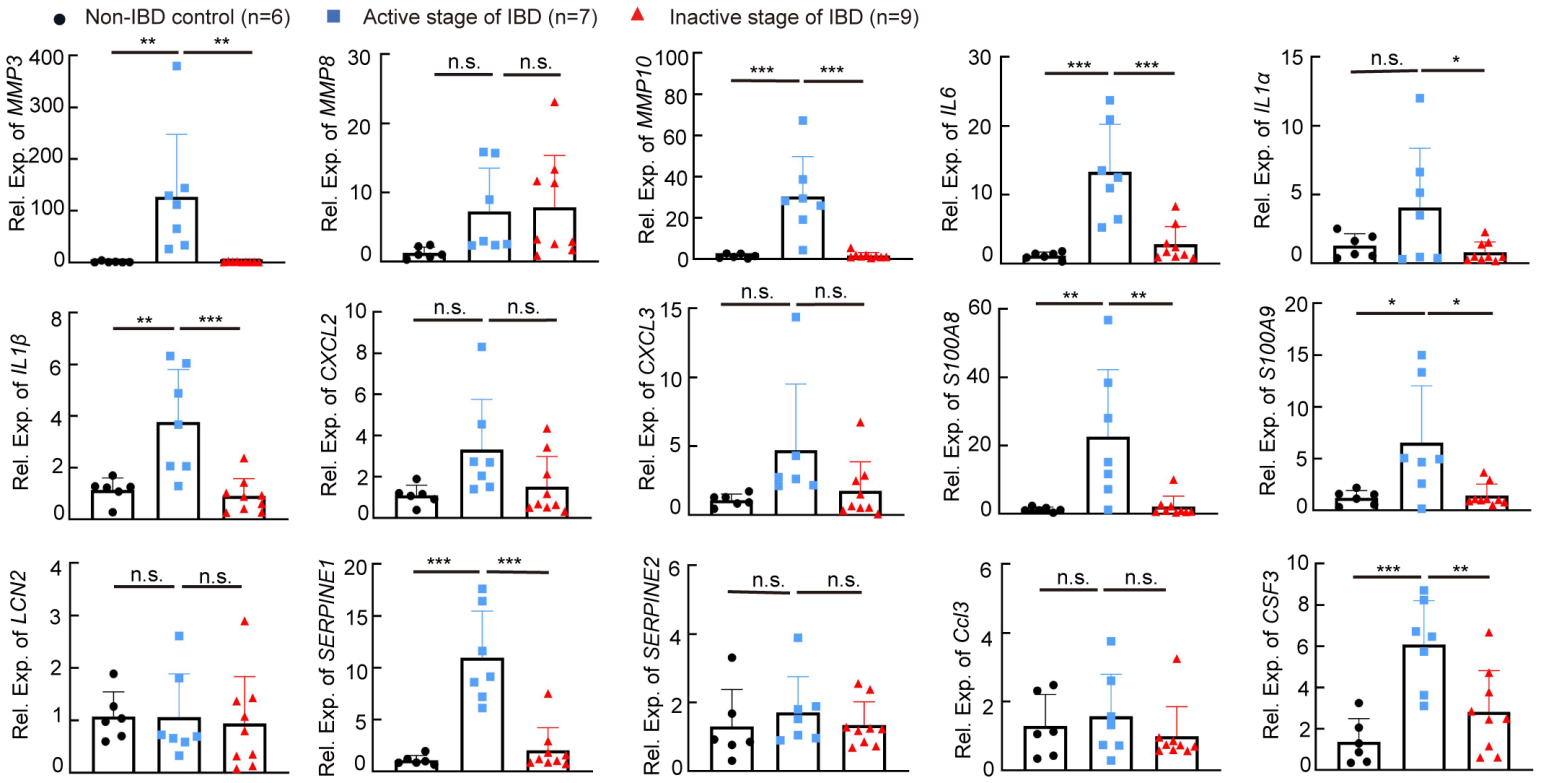
The expression level of IBD-related genes in human colon samples. The expression levels of *MMP3*, *MMP8*, *MMP10*, *IL6*, *IL1a*, *IL1b*, *CXCL2*, *CXCL3*, *S100A8*, *S100A9*, *LCN2*, *SERPINE1*, *SERPINE2*, *CCL3*, and *CSF3* in colon specimens of patients without IBD, or with IBD in the active stage or the inactive stage was measured by RT-qPCR; the results represent two independent experiments with triplicates of each sample. *n* = 6, 7, and 9. Data were shown as mean ± SD. *p < 0.05, **p < 0.01, ***p < 0.001, one-way ANOVA, n.s., no significance.

We next determined whether targets regulated by *miR-146a* increased in the colon of patients with IBD. Quantitative RT-PCR showed that the expression levels of *MMP3*, *MMP10*, *IL6*, *IL1B*, *S100A8*, *S100A9*, *SERPINE1*, and *CSF3* were higher in the colon of patients with active IBD than non-IBD subjects and patients with IBD in the inactive stage, and the expression level of *IL1A* was downregulated in patients with IBD in the inactive stage when compared to patients with active IBD (**Figure 9**). However, *MMP8*, *CXCL2*, *CXCL3*, *LCN2*, *SERPINE2*, and *CCL3* were not upregulated in the colon of patients with active IBD when compared to non-IBD subjects and patients with IBD in the inactive stage (**Figure 9**), but we should bear in mind that non-IBD subjects and patients with IBD in the inactive stage do not have health conditions, and these genes could be upregulated in these two groups of patients. Nevertheless, these findings suggest that *MMP3*, *MMP10*, *IL6*, *IL1B*, *S100A8*, *S100A9*, *SERPINE1*, *CSF3*, and *IL1A* are involved in active IBD.

## Discussion

**Figure 10:**
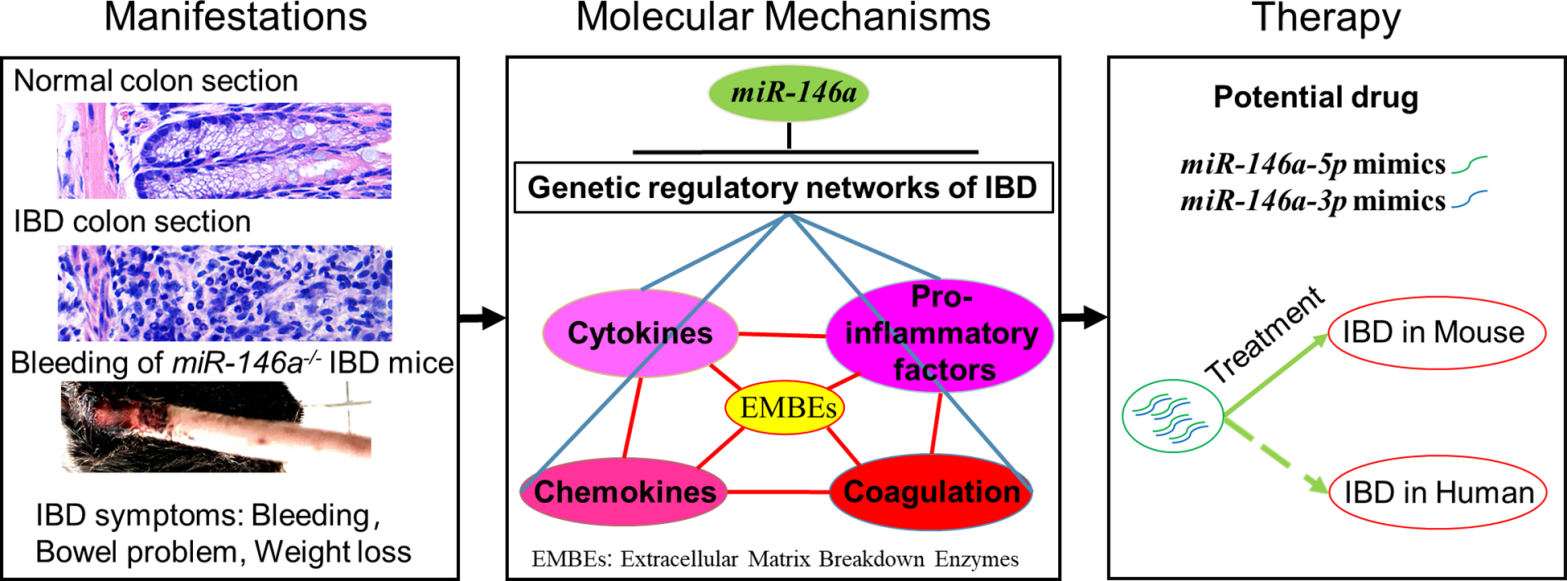
*MiR-146a* is a top regulator and potential drug of IBD. Deficiency of *MiR-146a* augments IBD due to de-repression of multiple genetic regulatory networks involved in IBD, and *miR-146a-5p* and *miR-146a-3p* mimics are potential drugs for IBD.

Here, we showed that the deficiency of *miR-146a* in mice with a C57BL/6N genetic background facilitates an immune response in the intestine to a normal environment and accelerates the severity of DSS-induced IBD, and that *MiR-146a-5p* and *miR-146a-3P* mimics are able to attenuate symptoms of DSS-induced IBD in mice (**Figure 10**). The underlying molecular mechanism is that *miR-146a* represses genetic regulatory networks of immune response, extracellular matrix breakdown enzymes, and coagulation in the intestine.

DSS induction in the *miR-146a-*deficient mice led to dramatic body weight loss, massive rectal bleeding, severe colitis, and damage to the intestine, mimicking the UC in humans, the main type of chronic IBD, which is characterized by recurrent and diffuse colorectal mucosa rupture, inflammation, and bleeding (3, 4). Moreover, *miR-146a* systematically represses close to 80% IBD genes, which cover multiple genetic regulatory networks of the cytokine family, chemokine family, pro-inflammatory factors, extracellular matrix breakdown enzymes, and coagulation. These findings suggest that *miR-146a* deficiency might represent major genetic susceptibilities for IBD, and that the *miR-146a* knock-out mouse is an ideal animal model for the induction of experimental IBD for drug screening and validation. Consistent with our data, Anzola et al. reported that the expression of *miR-146a* was increased in the colon of TNBS- or DSS-induced mouse models of colitis (42), and Runtsch et al. also demonstrated that the expression of *miR-146a* was higher in colonic biopsies from patients with UC than in those from healthy controls (27). However, conflicting data reported that the *miR-146a* knock-out mice with a C57BL/6 genetic background were resistant to DSS-induced colitis due to an enhanced intestinal barrier (27). The inconsistent response to DSS between our *miR-146a* knock-out mice with a C57BL/6N genetic background and the *miR-146a* knock-out mice with C57BL/6 is possible due to SNP variants on the direct targets of *miR-146a*, because it was reported that SNPs between C57BL/6J and C57BL/6N cause phenotypic variances (43), and that two *miR-146a* knock-out mouse lines were generated by using the same targeting vector, and only the *miR-146a* knock-out mice with a mixed C57BL/6 × 129/sv (but not C57BL/6) genetic background developed auto-immune diseases (25), which suggests that some pro-inflammatory targets are less repressed by *miR-146a* in the context of C57BL/6 genetic background. It seems that *miR-146a* represses inflammatory genes more than intestinal barrier-related genes in C57BL/6N mice, and it is the opposite in C57BL/6 mice. Thus, further investigation of the SNP variants in C57BL/6, 129/sv, and C57BL/6N mice would help develop a precise *miR-146a* mimic drug for subtypes of patients with IBD.

The RNA profile of the intestine derived from naïve *miR-146a* knock-out C57BL/6 mice unbiasedly demonstrated that *miR-146a* regulates many intestinal barrier-related genes, such as the Reg3 family (Reg3α, Reg3β, and Reg3γ), the Muc family (Muc3, Muc4, and Muc13), the Ceacam family (Ceacam1, Ceacam20, and Ceacam18), Epcam, claudins, occludin, and e-Cadherin, as well as a few inflammatory genes, such as Saa1, IL-18, and IL18bp (27). However, keep in mind that many pro-inflammatory genes are not expressed under physiological conditions. Thus, whether *miR-146a* would regulate more pro-inflammatory genes in the inflamed intestine of C57BL/6 mice is not known. Our RNA profiling data revealed the entire genetic regulatory network regulated by *miR-146a* in the inflammatory colon of C57BL/6N mice. Previous studies mostly focused on two direct targets of *miR-146a*, IRAK1 and TRAF6, to address the role of *miR-146a* in immune response (20, 44, 45). We found that, besides downregulating IRAK1 and TRAF6, *miR-146a* represses many other potential direct targets, such as *Il1a*, *Il11*, *Il5ra*, *Il1r2*, *Cxcl13*, *Cxcl5*, *Osm*, *Arg1*, *Igfbp5*, and *Vsig4.* Thus, *miR-146a* directly targets a number of critical immune response genes to systematically repress the majority of IBD genes induced by the toxic environment factor DSS. Because miRNA is a post-transcriptional regulator that may inhibit the protein translation of direct targets, instead of inducing degradation of target mRNAs under certain circumstances, the percentage of IBD-related genes repressed by *miR-146a* is likely higher than the corresponding gene number illustrated by RNA sequencing data. An example to support this speculation is that the mRNA levels of *Irak1*, *Traf6*, and p65 (RelA) were not significantly increased (**Supplementary Table S3**), but their protein expression was induced in DSS-treated *miR-146a* knock-out mice. Our data from DSS-treated *miR-146a* knock-out mice and RNA expression profile support that *miR-146a* is a top regulator of IBD and acts as a powerful brake and even a terminator of immune response.

MiRNAs are promising targets for IBD treatment (46–48). Our data demonstrated that the modified *miR-146a-5p* and *miR-146a-3p* mimics could attenuate DSS-induced IBD in both *miR-146a-*deficient and *WT* mice. We also found that the administration of *miR-146a-5p* mimics or *miR-146a-3p* mimics alone was insufficient to substantially relieve IBD in *miR-146a-*deficient mice (data not shown), indicating that *miR-146a-3p* also plays an important role in the regulation of IBD. Indeed, many predicted direct targets of *miR-146a-3p* were upregulated in DSS-treated *miR-146a-*deficient mice, e.g., p65 (RelA), which is predicted to be targeted by *miR-146a-3p*, not *miR-146a-5p*, increased in DSS-treated and untreated *miR-146a-*deficient mice. In line with our findings that show a significant increase in *miR-146a-3p* and an increasing trend of *miR-146a-5p* in patients with IBD compared to non-IBD patients, other laboratories reported that the SNP of *miR-146a* is associated with IBD (49, 50). For the purpose of late potential translational research, we used a human *miR-146a-3p* sequence instead of a mouse *miR-146a-3p* sequence (one nucleotide differs in seed sequence between them, see **Supplementary Table S2**) in the treatment experiments of mouse models; thus, it is believed that a better efficacy would be achieved if the sequence of the *miR-146a-3p* mimic matches the applied species. The lower efficacy of the *miR-146a-3p* mimics relative to endogenous *miR-146a-3p* explains why *miR-146a-*deficient mice, which had no endogenous *miR-146a-3p*, required more *miR-146a-3p* mimics than WT mice, which had endogenous *miR-146a-3p*. Given the sequences of mature *miR-146a-5p* and *miR-146a-3p* and that the binding sites on their target genes are highly conserved among mouse, rat, and human (51, 52), our findings suggest that *miR-146a-5p* and *miR-146a-3p* mimics confer great promise for IBD treatment. Note that the effect of 80 pg/g of *miR-146a-5p* mimics and 80 pg/g of *miR-146a-3p* mimics was not as good as the dosage of 40 pg/g of *miR-146a-5p* mimics and 80 pg/g of *miR-146a-3p* mimics (data not shown). This suggests that the higher dose of *miR-146a-5p* mimics could bring side effects, since *miR-146a* also represses the intestinal barrier function (27). Another possible risk from *miR-146a* mimics treatment could be an increase in infection as innate immunity was repressed. Thus, the dose of *miR-146a* mimics is critical for the treatment of IBD.

We also found that the administration of *miR-146a* mimics only via gavage did not significantly relieve DSS-induced IBD in mice (data not shown), and that only the combination of gavage and intraperitoneal injection did. Intraperitoneal injection enables high concentration of *miR-146a* mimics in blood to regulate the function of immune cells, which is consistent with the previous reports that show that *miR-146a* deficiency affects the function of T cells through STAT1, NF-κB, TNF, IRAK1, and TRAF6 (53–55) and B cells via regulating the germinal center response and the secretion of IL1 and IL6 (56–59). Thus, our data suggest that the inhibition of both intestinal inflammation and activation of immune cells is necessary for *miR-146a* mimics to efficiently treat IBD.

In conclusion, *miR-146a* acts as a top regulator to systematically repress multiple genetic regulatory networks involved in the immune response of the intestine to environment factors, and the combinatory treatment using *miR-146a-5p* and *miR-146a-3p* mimics attenuates DSS-induced IBD in mice through downregulating multiple genetic regulatory networks that were increased in colon tissue from patients with IBD. Our data suggest that the *miR-146a* knock-out mice with a C57/BL6N genetic background treated with DSS can be a useful model for colitis studies, and that *miR-146a-5p* and *miR-146a-3p* mimics may be potential therapeutic drugs for IBD.

## Data availability statement

The datasets presented in this study can be found in online repositories. The names of the repository/repositories and accession number(s) can be found below: GSE247433 (GEO).

## Ethics statement

The studies involving humans were approved by the Ethics Committee of The Third Xiangya Hospital. The studies were conducted in accordance with the local legislation and institutional requirements. Written informed consent for participation in this study was provided by the participants’ legal guardians/next of kin. The animal study was approved by Tongji University ethical review panel. The study was conducted in accordance with the local legislation and institutional requirements.

## Author contributions

FZ: Writing – original draft, Software, Methodology, Investigation, Formal analysis, Data curation. TY: Writing – original draft, Methodology, Investigation, Formal analysis. MN: Writing – review & editing, Methodology, Formal analysis. YLiu: Writing – review & editing, Methodology, Formal analysis. WX: Writing – review & editing, Methodology. YF: Writing – review & editing, Methodology. TW: Writing – review & editing, Methodology. MZ: Writing – review & editing, Methodology. RX: Writing – review & editing, Methodology. RQ: Writing – review & editing, Methodology. YLi: Writing – review & editing, Methodology. MS: Writing – review & editing. JL: Writing – review & editing. LT: Writing – review & editing, Resources, Methodology, Investigation. QZ: Writing – review & editing. XY: Writing – review & editing, Supervision. CP: Writing – review & editing, Writing – original draft, Supervision, Project administration, Funding acquisition, Conceptualization.
