# Supplementary material for: MiR-146a alleviates inflammatory bowel disease in mice through systematic regulation of multiple genetic networks
Source: Front Immunol. 2024 May 10;15:1366319. doi: 10.3389/fimmu.2024.1366319 (PMC11116640; doi:10.3389/fimmu.2024.1366319)
Supplement: Supplementary file 1 [file DataSheet_1.pdf]

Supplementary Material

This PDF file includes:

Figures S1 to S5 and legends
Table S1 to S3, and Table S4 to S6 legends

Other Supplementary Materials for this manuscript includes the

following: Table S4 to S6, excel files

Supplementary figures and figure legends

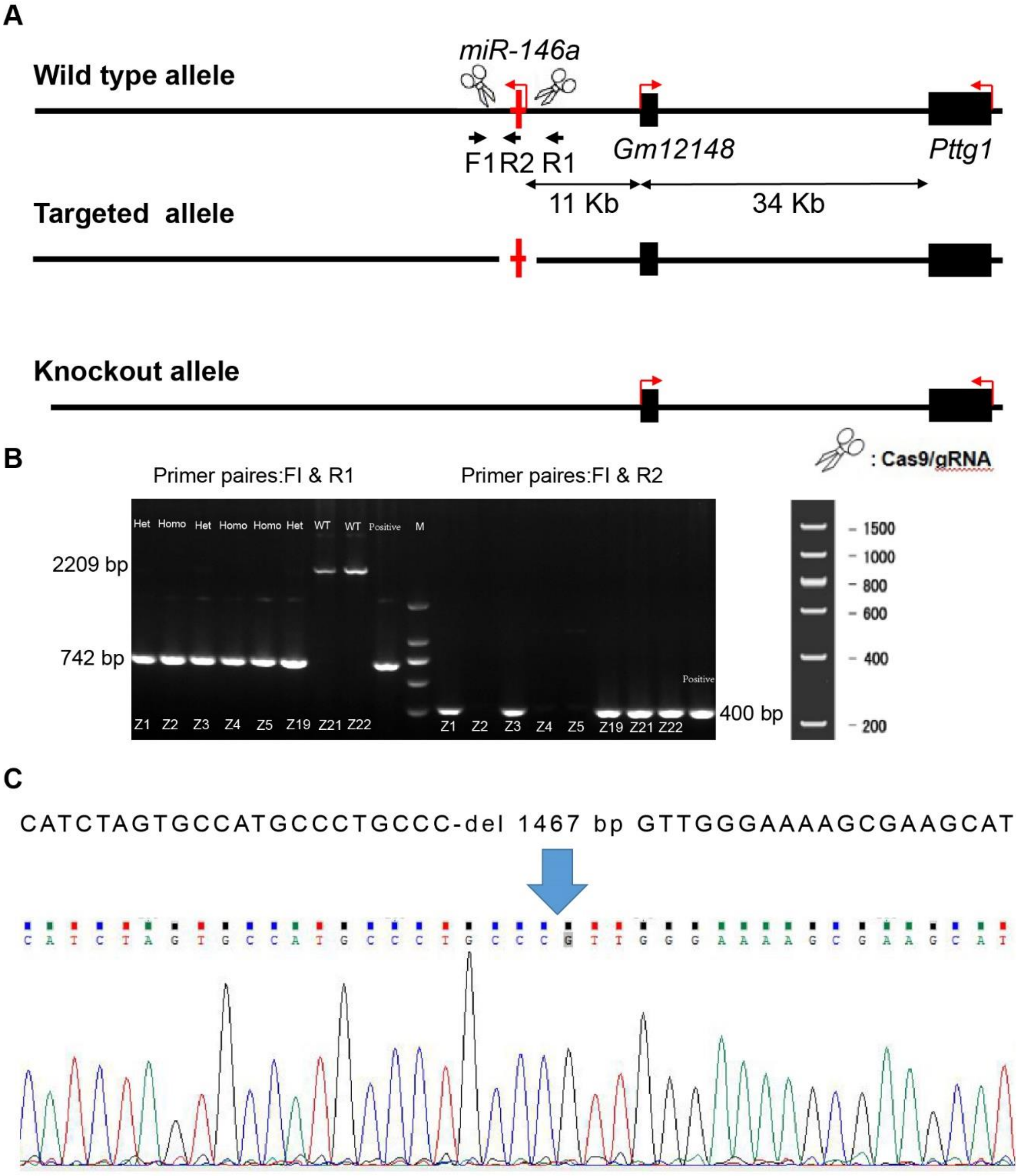

Figure S1. Generation of *miR-146a* knockout mice.

(A) Schematic illustration of Cas9/gRNA-mediated gene-targeting for the *miR-146a* genomic locus， including the wild type (*WT*) allele, the targeted allele and knockout allele.

(B) PCR (location of primers F1, R1 and R2 showed in A) was used to genotype the mice carrying *WT* allele and/or knockout allele, with primer pair F1&R1 amplifying a 400 bp PCR product from *WT* allele, while primer pair F1&R2 amplifying a 2209 bp and a 742 bp PCR products from *WT* allele and *miR-146a* knockout allele, respectively.

(C) Sanger sequencing of knockout allele showed that a 1467 bp DNA fragment

containing *miR-146a* gene was deleted in the *miR-146a* KO mice. The targeted deletion didn't cause mutations in *miR-146a* neighbor genes.

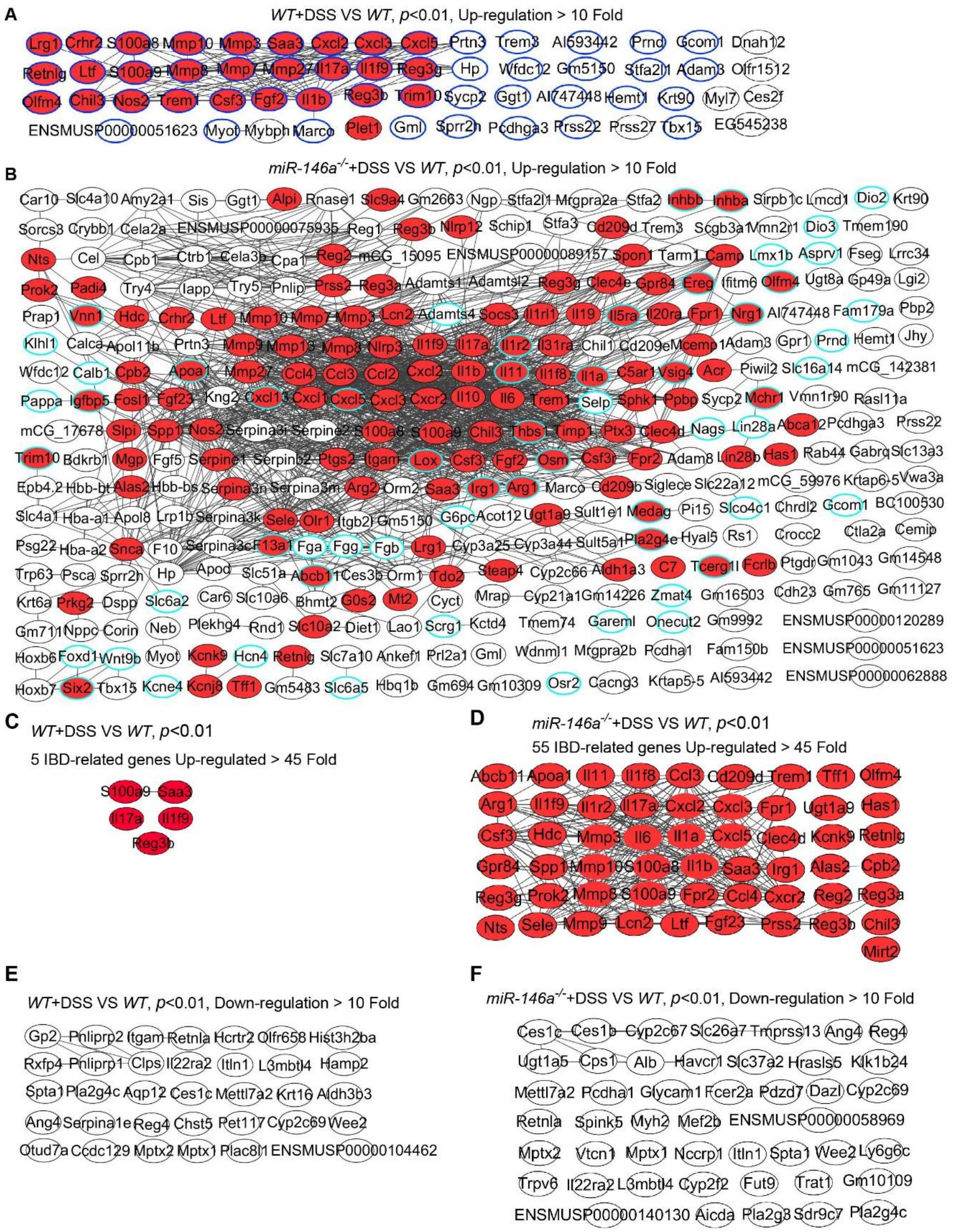

Figure S2. The change of genetic regulatory network in colon of *WT* and *miR-146a$^{-/-}$* mice under DSS treatment.

(A) The functional interaction network of up-regulated genes in colon of DSS-treated *WT* mice was analyzed using String database APP in Cytoscape. Out of the 71 up-regulated genes, 58 with or without predicted connections (gray lines, inclusion criteria: up-regulated fold ≥ 10 and *p* value <0.01) were shown. Blue circles indicated up-regulated genes in the colon of DSS-treated *miR-146a$^{-/-}$* mice, while red marked 28

IBD-related genes.

(B) The functional interaction network of up-regulated genes in colon of *miR-146a*$^{-/-}$ mice, compared to naïve *WT* mice, was analyzed using String database APP in Cytoscape. Out of the 381 up-regulated genes, 327 with or without predicted connections (gray lines, inclusion criteria: up-regulated fold ≥ 10 and p value <0.01) were shown. Cyan circles indicated direct target genes of *miR-146a* predicted by TragetScan8.0, while red marked IBD-related genes.

(C, D) Five IBD-related genes was up-regulated by more than 45 folds in colon of DSS-treated *WT* mice when compared to naïve *WT* mice (C), but much more (55) IBD-related genes were up-regulated by more than 45 folds in colon of DSS-treated *miR-146a*$^{-/-}$ mice when compared to naïve *WT* mice.

(E) The functional interaction network of down-regulated genes in colon of DSS-treated *WT* mice was analyzed using String database APP in Cytoscape. Out of the 45 down -regulated genes, 34 with or without predicted connections (gray lines, inclusion criteria: down-regulated fold ≥ 10 and p value <0.01) were shown.

(F) The functional interaction network of down-regulated genes in colon of *miR-146a*$^{-/-}$ mice, compared to naïve *WT* mice, was analyzed using String database APP in Cytoscape. Out of the 64 down-regulated genes, 46 with or without predicted connections (gray lines, inclusion criteria: up-regulated fold ≥ 10 and p value <0.01) were shown.

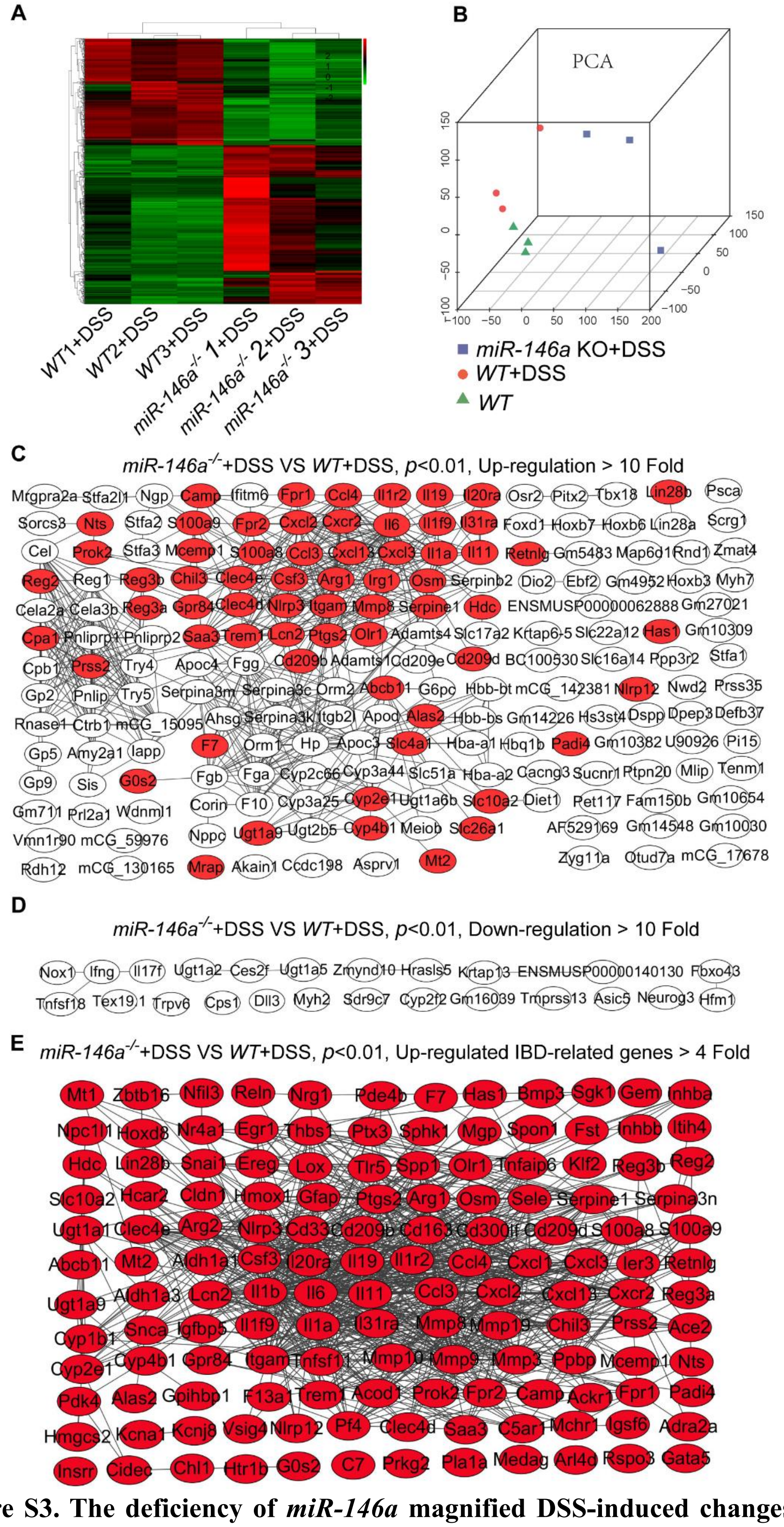

Figure S3. The deficiency of *miR-146a* magnified DSS-induced changes of gene expressions in colon.

(A) Heat map of differentially expressed genes in colons between DSS-treated *miR-*

146a$^{-/-}$ mice and DSS-treated *WT* mice.

(B) Principal component analysis of mRNA profile of colon from *WT* mice, DSS-treated *miR-146a*$^{-/-}$ mice and DSS-treated *WT* mice.

(C) The functional interaction network of up-regulated genes in colon of *miR-146a*$^{-/-}$ mice, when compared to DSS-treated *WT* mice, was analyzed using String database APP in Cytoscape. Out of the 225 up-regulated genes, 189 with or without predicted connections (gray lines, inclusion criteria: down-regulated fold ≥ 10 and *p* value <0.01) were shown.

(D) The functional interaction network of down-regulated genes in colon of *miR-146a*$^{-/-}$ mice, when compared to DSS-treated *WT* mice, was analyzed using String database APP in Cytoscape. Out of the 41 down -regulated genes, 24 with or without predicted connections (gray lines, inclusion criteria: down-regulated fold ≥ 10 and *p* value <0.01) were shown.

(E) The functional interaction network of up-regulated IBD-related genes in colon of *miR-146a*$^{-/-}$ mice, when compared to DSS-treated mice, was analyzed using String database APP in Cytoscape. Out of the 141 up-regulated IBD-related genes, 139 with or without predicted connections (gray lines, inclusion criteria: down-regulated fold ≥ 10 and *p* value <0.01) were shown.

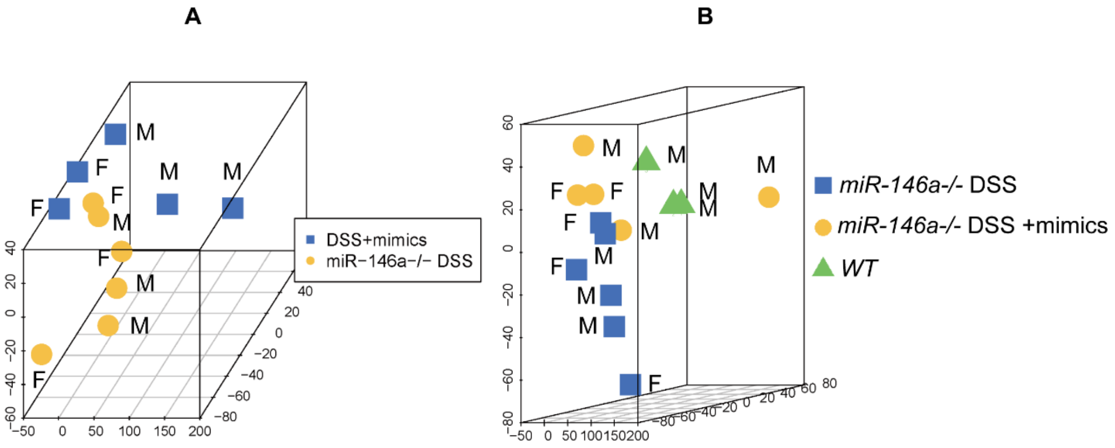

Figure S4. ***MiR-146a*** **mimics treatment globally pulled flamed expression profile back towards health status.**

A. Principal analysis of mRNA expression profile of colon from mimics-treated and saline-treated *miR-146a*$^{-/-}$ mice. B. Principal analysis of mRNA expression profile of colon from *WT* mice and mimics-treated and saline-treated *miR-146a*$^{-/-}$ mice.F: female, M: male.

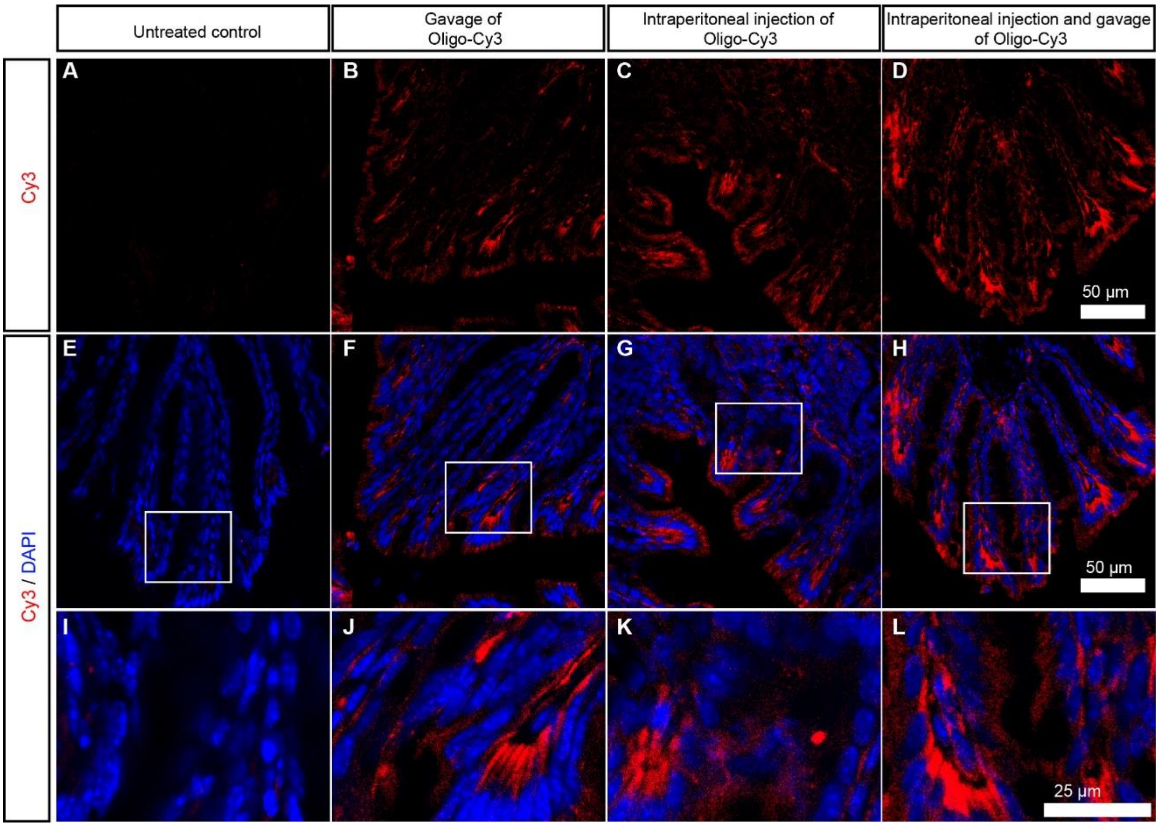

Figure S5. The uptake of *miR-146a* mimics by colon in *WT* mice.

(A-H) Fluorescence images of DAPI (blue) stained colon section from untreated control *WT* mice (A, E) and *WT* mice with gavage of Cy3-conjugated *miR-146a* mimics (B, F), or with intraperitoneal injection of Cy3-conjugated *miR-146a* mimics (C, G), or combination gavage and intraperitoneal injection of Cy3-conjugated *miR-146a* mimics (D, H) 4 hours after administration. (I-L) I-L are high magnification view of the boxed area in E-H. The images represent the absorption results from three technical repeats of the staining, n=4,4,4,4.

Table S1 to S3

Table S1: Information of IBD patients

Sample ID	Age	Gender	Diagnosis	Complications	miR-146a 5P	miR-146a 3P	Medications	CPR(mg/L)(0-6)	WBC (*10^9g /L)(3.5-9.5)
1	20	Female	CD (A2LB1, Active)	1. Moderate anemia<br>2. Hypoproteinemia	1.36	4.01	Adalimumab	<5.00	5.77
2	39	Male	CD (A2L1B1, Active)	Chronic non-atrophic gastritis with erosion	2.40	2.80	Isoniazid, Compound glutamine, Bifid triple viables, Infliximab, Ustekinumab	<5.00	4.14
3	33	Male	CD (A2L1B1, ACTIVE)	No	0.53	5.44	Infliximab, Ustekinumab	6.39	9.05
4	30	Male	CD (A2L3B2, Active)	1. Small nodules in both lungs<br>2. Muller's cyst of the prostate	2.44	5.04	Infliximab	6.05	6.72
5	18	Male	CD (A2L3B1, Active)	No	2.97	4.69	Ustekinumab	<5.00	3.59
6	36	Male	CD (A2L3B2P, Active)	1. Chronic non-atrophic gastritis<br>2. Left kidney cyst	1.66	4.11	Infliximab	6.16	6.26
7	28	Male	CD (A2L1B2,	1. Gastric antral ulcer (H1) 2. Chronic	1.19	4.56	Ustekinumab	<5.00	4.79

			Active)	non-atrophic gastritis 3. Ileostomy resection + intestinal adhesiolysis + cecalctomy 4. Gallstones					
8	53	Male	CD (A3L3B2) (Clinical remission)	1. Chronic non-atrophic gastritis 2. Lung nodules 3. Liver cysts 4. Kidney cysts 5. Chronic prostatitis 6. Schistosomiasis	2.50	3.39	Azathioprine, Adalimumab, Infliximab	<5.00	3.49
9	47	Male	CD (A3L1B3) (Clinical remission)	1. Partial resection of the small intestine + postoperative release of intestinal adhesions<br>2. Nutritional risks	0.97	0.79	Ustekinumab	<5.00	3.03
10	35	Female	CD (Clinical remission)	No	3.60	3.82	Infliximab, Azathioprine	<5.00	3.73
11	21	Female	CD ((A2L3Bp, clinical remission)	1. Left kidney cancer surgery<br>2. Duodenal ulcer 3. Colorectal multiple polyps	3.39	4.02	Compound Glutamine enteric-coated capsules, Bifidus triple viable bacteria powder, Mesalazine, Ubimex tablets, Ustekinumab	<5.00	7.15
12	47	Male	UC (mayo 1 point) (Clinical remission)	1. Chronic non-atrophic gastritis 2. Bilateral lung nodules LU-RADS class 4A) 3. Postoperative nasal polyps 4. Anxiety state	2.00	6.40	Mesalazine, Compound glutamine, Vedolizumab	<5.00	5.21
13	35	Male	CD (clinical remission)	1. After jejunal stricture incision<br>2. Chronic non-atrophic gastritis<br>3. Proctitis	1.24	6.65	Infliximab	<5.00	10.97

14	51	Female	UC（clinical remission)	1. Rectal polyps 2. Internal hemorrhoids 3. Left kidney cysts 4. Bilateral lung nodules LU-RADS class 2 (consider benign nodules)	2.32	3.49	Mesalazine	<5.00	10.97
15	38	Male	CD（clinical remission）	1. Fatty liver 2. Right kidney cyst	Excludec (too high)	Excludec (too high)	Infliximab	<5.00	4.98
16	61	Male	UC（clinical remission）	1.Type 2 diabetes mellitus with peripheral neuropathy 2.Hypokalemia 3.Impaired liver function (drug-induced) 4.Gastric varices 5.Esophageal papilloma 6.Chronic non-atrophic gastritis 7.Duodenal bulb mucosal bulge: external pressure may be 8.Multiple polyps in the large intestine 9.Multiple polyps in the colon	1.28	2.67	Mesalazine	<5.00	6.16
Information of Non-IBD patients									
1	30	Female	Non-IBD	Chronic colitis (3-4cm mucosa from the anus)	2.09	1.05	NA	NA	NA
2	40	Female	Non-IBD	Chronic enteritis (terminal ileum)	1.71	0.62	NA	NA	NA
3	68	Male	Non-IBD	Chronic enteritis (terminal ileum)	0.89	0.62	NA	NA	NA
4	38	Male	Non-IBD	Chronic enteritis (terminal ileum)	0.80	0.42	NA	NA	NA
5	42	Male	Non-IBD	Chronic enteritis (terminal ileum)	0.98	3.38	NA	NA	NA
6	54	Male	Non-IBD	Chronic enteritis (terminal ileum)	0.41	1.73	NA	NA	NA

Table S2: The sequence of *miR-146a* mimics

**Mimics**	**Sense(5'-3')**
MiR-146a-5p	mU*mG*mAmGmAmAmCmUfGmAmAmUmUCmCmAmUmGmGmGmU*mU*+A
MiR-146a-3p	mC*mC*mUmGmUmGAmAfAmUmUmCmAmGmUmUfCfUfUmC*fA*+G
MiR-146a-5p-Cy3	CY3-mU*mG*mAmGmAmAmCmUfGmAmAmUmUCmCmAmUmGmGmGmU*mU*+A
MiR-146a-3p- Cy3	CY3-mC*mC*mUmGmUmGAmAfAmUmUmCmAmGmUmUfCfUfUmC*fA*+G

Explanation of writing format for bases and modifications in sequences

(1) 2' O-methyl RNA base："m_", e.g: mA, mU.

(2) 2'-Fluoro RNA base: "f_", e.g:fA, fU.

(3) Thiomodification between two bases: "*".

(4) LNA-A: +A.

(5) LNA-G:+G.

Table S3: The PCR primers

Gene	Primers
m*Mmp3*	F:5’-ACTCAAGGGTGGATGCTGTC-3’
	R:5’-TTGGGTCAAATTCCAACTGCG-3’
m*Mmp8*	F:5’-CACACTCCGTGGGGAGATTT-3’
	R:5’-CCTGAAGACCGTTGGGTAGG-3’
m*Mmp10*	F:5’-CGCTGAGAGGGGAAGTCCTA-3’
	R:5’-AGAGTGGGCCAAAATGCTGA-3’
m*Il6*	F:5’-CCACTTCACAAGTCGGAGGCTTA-3’<br>R:5’-GCAAGTGCATCATCGTTGTTCATAC-3’
m*Il1a*	F:5’-ACAGGTAGTGAGACCGACCT-3’<br>R:5’-GGTGCACCCGACTTTGTTCT-3’
m*Il1b*	F:5’-TCCAGGATGAGGACATGAGCAC-3’<br>R:5’-GAACGTCACACACCAGCAGGTTA-3’
m*Cxcl2*	F:5’-CAGTGCCTCCAACAAGCTTCC-3’<br>R:5’-CCTGTAGCCTGGTGGTTGGT-3’
m*Cxcl3*	F:5’-ATCCCAACGGTGTCTGGATG-3’<br>R:5’-GCAAGTAGATGCAATTATACCCGT-3’
m*S100a8*	F:5’-CAGCTGACACTTAGCCTCACA-3’<br>R:5’-CTTCTCCAGTTCAGACGGCA-3’
m*S100a9*	F:5’-CCTTGAAGAGCAAGAAGATGGC-3’<br>R:5’-TCAGGGTGTCCTTCCTTCCT-3’
m*Lcn2*	F:5’-GGAACGTTTCACCCGCTTTG-3’<br>R:5’-CCACACTCACCACCCATTCA-3’
m*Serpine1*	F:5’-CCGATGGGCTCGAGTATGAC-3’<br>R:5’-TTCTCAAAGGGTGCAGCGAT-3’
m*Serpine2*	F:5’-CCGTAGCCTCTTCGTGTGTT-3’<br>R:5’-CAGTAGTGACAGGTACGCGG-3’

m*Ccl3*	F:5’-CATATGGAGCTGACACCCCG-3’<br>R:5’-GAGCAAAGGCTGCTGGTTTC-3’
m*Saa3*	F:5’-GACAGAAGCCACTCACCTGG-3’<br>R:5’-GCTGTCAACTCCCAGGATCAA-3’
m*Csf3*	F:5’-CGGGGCCTTGATGGCTTTAT-3’<br>R:5’-GGATTCTGGGTGATCTGGGC-3’
m*mir-146-3P*	F:5’-CCTGTGAAATTCAGTTCTTCAGTT-3’
m*mir-146-5p*	F:5’-TGAGAACTGAATTCCATGGGTTTT-3’
mir Rev2	F:5’-TGTTAGGTTCTGTGATTGAGGCA-3’
m*Gapdh*	F:5’-AGGTCGGTGTGAACGGATTTG-3’
	R:5’-TGTAGACCATGTAGTTGAGGTCA-3’

Gene	Primers
h*MMP3*	F:5’-TCACTCACAGACCTGACTCG-3’
	R:5’-GTCAGGGGGAGGTCCATAG-3’
h*MMP8*	F:5’-ACAAAAACTGTTCAGGACTACCT-3’
	R:5’-TTGGCTTCCCCGTCACATTC-3’
h*MMP10*	F:5’-CCTTGTGCTGTTGTGTCTGC-3’
	R:5’-ACTTTTCTAGGTATTGCTGGGCA-3’
h*IL6*	F:5’-AGCCCACCGGGAACGAAA-3’<br>R:5’-CCGAAGGCGCTTGTGGAG-3’
h*IL1A*	F:5’-GCGTTTGAGTCAGCAAAGAAGT-3’<br>R:5’-CAGAGACAGATGATCAATGGAGGA-3’
h*IL1B*	F:5’-TGATGGCTTATTACAGTGGCAA-3’<br>R:5’-GGTGGTCGGAGATTCGTAGC-3’
h*CXCL2*	F:5’-GCTTGTCTCAACCCCGCATC-3’<br>R:5’-TCTGGTCAGTTGGATTTGCCATTTT-3’
h*CXCL3*	F:5’-ATCGAAAAGATACTGAACAAGGGGA-3’

	R:5'-ATTTTCAGCTCTGGTAAGGGCA-3'
h*S100A8*	F:5'-TTTCAGAAGACCTGGTGGGG-3'<br>R:5'-CCCTGTAGACGGCATGGAAA-3'
h*S100A9*	F:5'-CCTCGGCTTTGACAGAGTG-3'<br>R:5'-CACCAGCTCTTTGAATTCCCC-3'
h*LCN2*	F:5'-AGTGCACAGGTGCCGC-3'<br>R:5'-TTTAGCAGACAAGGTGGGGCT-3'
h*SERPINE1*	F:5'-ACCTCTGAGAACTTCAGGATGC-3'<br>R:5'-CACAGCAGACCCTTCACCAA-3'
h*SERPINE2*	F:5'-CGAGCGCGGTCGTCCT-3'<br>R:5'-CCCGTGTTGGAGCCTAGTTC-3'
h*CCL3*	F:5'-CCGTCACCTGCTCAGAATCAT-3'<br>R:5'-GCGTGTCAGCAGCAAGTGAT-3'
h*CSF3*	F:5'-CTCCAGGAGAAGCTGTGTGCC-3'<br>R:5'-AGGGGATGCCCAGAGAGTGT-3'
h*mir-146-3P*	F:5'-CCTCTGAAATTCAGTTCTTCAGTT-3'
m*mir-146-5p*	F:5'-TGAGAACTGAATTCCATGGGTTTT-3'
mir Rev2	F:5'-TGTTAGGTTCTGTGATTGAGGCA-3'
h*GAPDH*	F:5'-TGACAACTTTGGTATYCGTGGAAGG-3'<br>R:5'-AGGCAGGGATGATGTTCTGGAGAG-3'

Table S4 to S6 legends

Table S4. This Microsoft Excel file includes three datasheets. The first shows expression profile in colons from DSS-treated *WT* and untreated *WT* mice; the second displays gene expression profile in colons from DSS-treated *miR-146a*$^{-/-}$ and untreated *WT* mice; and the third presents gene expression profile in colons from DSS-treated *miR-146a*$^{-/-}$ and DSS-treated *WT* mice.

Table S5. The list of differentially expressed genes in comparison of DSS-treated *WT* mice versus untreated *WT* mice (sheet1), DSS-treated *miR-146a*$^{-/-}$ mice versus untreated *WT* mice (sheet2) and DSS-treated *miR-146a*$^{-/-}$ mice versus DSS-treated *WT* mice (sheet3).

Table S6. This Microsoft Excel file includes three datasheets. The first one shows gene the expression profile in colons from saline + DSS-treated *miR-146a*$^{-/-}$ and *miR-146a* mimics + DSS -treated *miR-146a*$^{-/-}$ mice; the second and third presents the list of down-regulated and up-regulated genes by more than 2-fold in colons of *miR-146a* mimics + DSS -treated *miR-146a*$^{-/-}$ mice compared to saline + DSS-treated *miR-146a*$^{-/-}$ mice, respectively.
